# A biallelic mutation in CACNA2D2 associated with developmental and epileptic encephalopathy affects calcium channel‐dependent as well as synaptic functions of α_2_δ‐2

**DOI:** 10.1111/jnc.16197

**Published:** 2024-08-19

**Authors:** Sabrin Haddad, Cornelia Ablinger, Ruslan Stanika, Manuel Hessenberger, Marta Campiglio, Nadine J. Ortner, Petronel Tuluc, Gerald J. Obermair

**Affiliations:** ^1^ Institute of Physiology Medical University Innsbruck Innsbruck Austria; ^2^ Division of Physiology Department of Pharmacology, Physiology, and Microbiology Karl Landsteiner University of Health Sciences Krems Austria; ^3^ Department of Pharmacology and Toxicology University of Innsbruck Innsbruck Austria

**Keywords:** auxiliary subunit, calcium current, epilepsy, neurodevelopmental disorders, trans‐synaptic function, voltage‐gated calcium channels

## Abstract

α_2_δ proteins serve as auxiliary subunits of voltage‐gated calcium channels and regulate channel membrane expression and current properties. Besides their channel function, α_2_δ proteins regulate synapse formation, differentiation, and synaptic wiring. Considering these important functions, it is not surprising that CACNA2D1‐4, the genes encoding for α_2_δ‐1 to ‐4 isoforms, have been implicated in neurological, neurodevelopmental, and neuropsychiatric disorders. Mutations in CACNA2D2 have been associated with developmental and epileptic encephalopathy (DEE) and cerebellar atrophy. In our present study, we performed a detailed functional characterization of the p.R593P mutation in α_2_δ‐2, a homozygous mutation previously identified in two siblings with DEE. Importantly, we analyzed both calcium channel‐dependent as well as synaptic functions of α_2_δ‐2. Our data show that the corresponding p.R596P mutation in mouse α_2_δ‐2 drastically decreases membrane expression and synaptic targeting of α_2_δ‐2. This defect correlates with altered biophysical properties of postsynaptic Ca_V_1.3 channel but has no effect on presynaptic Ca_V_2.1 channels upon heterologous expression in tsA201 cells. However, homologous expression of α_2_δ‐2_R596P in primary cultures of hippocampal neurons affects the ability of α_2_δ‐2 to induce a statistically significant increase in the presynaptic abundance of endogenous Ca_V_2.1 channels and presynaptic calcium transients. Moreover, our data demonstrate that in addition to lowering membrane expression, the p.R596P mutation reduces the trans‐synaptic recruitment of GABA_A_ receptors and presynaptic synapsin clustering in glutamatergic synapses. Lastly, the α_2_δ‐2_R596P reduces the amplitudes of glutamatergic miniature postsynaptic currents in transduced hippocampal neurons. Taken together, our data strongly link the human biallelic p.R593P mutation to the underlying severe neurodevelopmental disorder and highlight the importance of studying α_2_δ mutations not only in the context of channelopathies but also synaptopathies.

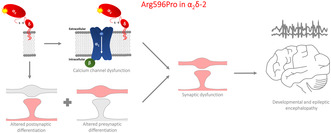

## Graphical abstract

α_2_δ auxiliary subunits of voltage‐gated calcium channels modulate the calcium currents and have emerged as regulators of synaptic functions. Considering these important functions, it is not surprising that mutations of α_2_δ‐2 are associated with developmental and epileptic encephalopathy (DEE). However, whether an altered calcium channel modulation, synaptic mechanism, or both underlie the disease phenotype is not known. Here, we functionally characterized a homozygous missense mutation identified in two siblings with DEE. Our study strongly links the mutation to the underlying disorder and demonstrates that disruption in both channel‐dependent as well as pre‐ and postsynaptic functions of α_2_δ‐2 can contribute to the pathophysiological mechanism.

**Abbreviations**
Ca^2+^
: calcium
CACNA2D1–4: calcium channel auxiliary subunit alpha2delta‐coding genes
Ca_V_
: voltage‐gated calcium channels
CNS: central nervous system
DEE: developmental and epileptic encephalopathy
DIV: days in vitro
GABA_A_R: GABA_A_ receptor
HA: hemagglutinin
mEPSCs: miniature excitatory postsynaptic currents
MIDAS: metal ion‐dependent adhesion site
RRID: Research Resource Identifier
VGCC: voltage‐gated calcium channel
vGLUT1: vesicular glutamate transporter 1
VWA: von Willebrand factor type A domain
WT: wild type

## 1 INTRODUCTION

Calcium (Ca^2+^) entry into excitable cells is tightly regulated by voltage‐gated Ca^2+^ channels (VGCCs, Ca_V_). Neuronal channels of the Ca_V_1 and Ca_V_2 families are multimeric complexes consisting of pore‐forming α_1_ and two auxiliary subunits, β and α_2_δ. Four genes encode for α_2_δ subunits (CACNA2D1‐4) out of which three isoforms (α_2_δ‐1‐3) are highly expressed in the central nervous system (CNS) (Schlick et al., 2010). The classical roles of α_2_δ proteins as auxiliary subunits of VGCCs, regulating the functional membrane expression and modulating the Ca^2+^ currents, are widely studied (reviewed in Dolphin, 2016). Recently, all four α_2_δ isoforms were recognized as important regulators of synaptic functions and these functions may be partially or entirely independent of the Ca^2+^ channel complex (reviewed in Dolphin & Obermair, 2022; Geisler et al., 2015). For example, loss of presynaptic α_2_δ subunits in murine cultured hippocampal neurons disrupted both pre‐ and postsynaptic differentiation, indicating that α_2_δ isoforms are necessary for glutamatergic synapse formation and trans‐synaptic differentiation (Schopf et al., 2021). Moreover, altered expression levels of presynaptic α_2_δ‐1 and α_2_δ‐2 splice variants induce aberrant synaptic wiring and change the postsynaptic molecular composition by a trans‐synaptic mechanism (Ablinger et al., 2022; Geisler et al., 2019). This emphasizes the importance of α_2_δ proteins in brain connectivity and suggests that aberrant expression of α_2_δ proteins may affect the excitatory–inhibitory balance. Therefore, it is not surprising that α_2_δ proteins have been implicated in various neurological disorders (reviewed in Ablinger et al., 2020; Ablinger et al., 2024; Hessenberger et al., 2023).

However, whether aberrant α_2_δ protein functions cause such defects by altered Ca^2+^ channel modulation, synaptic mechanism, or both is largely unknown. Several genes encoding VGCC subunits have been associated with epileptic encephalopathy, including CACNA1A (Epi, 2016; Epi et al., 2013; Niu et al., 2022), CACNA1C (Bozarth et al., 2018), CACNA1E (Helbig et al., 2018), CACNA2D1 (Dahimene et al., 2022), and CACNA2D2 (Table 1). The α_2_δ‐2 isoform is highly expressed in the cerebellum and is relevant for the structure and function of cerebellar synapses (Beeson et al., 2020; Beeson et al., 2021). Besides α_2_δ‐1, it is one of the targets of the highly prescribed anti‐epileptic drugs pregabalin and gabapentin (Gong et al., 2001). To date, five cases of infantile‐onset epilepsy and cerebellar atrophy linked to bi‐allelic mutations in CACNA2D2 have been reported in unrelated families (Table 1; Butler et al., 2018; Edvardson et al., 2013; Pippucci et al., 2013; Punetha et al., 2019). The clinical presentation of all patients is strikingly similar to the phenotypes of ducky mutant mice, carrying a CACNA2D2 loss‐of‐function mutation, and α_2_δ‐2 knockout mice (Barclay et al., 2001; Brodbeck et al., 2002; Donato et al., 2006; Ivanov et al., 2004), strengthening the hypothesis that abnormal α_2_δ‐2 expression or function may underlie the epileptic phenotype for these patients. However, as of today, in‐depth mechanistic analysis of these mutations is still missing and, if studied, limited to the so‐called “channelopathies,” essentially describing the consequences of α_2_δ mutations on Ca^2+^ channel functions (Edvardson et al., 2013).

**TABLE 1:** Previously reported cases of infantile‐onset epilepsy with bi‐allelic mutations in CACNA2D2.

**Publication**	Genetic alteration	Patient	Common main symptoms	Seizure onset (month)	Functional characterization
Edvardson et al., 2013	Homozygous c.3199A > g (p.L1040P)	Three affected siblings (2 Female, 1 Male)	Infantile‐onset epilepsy Global developmental Delay Cerebellar atrophy	1–2	Failed to increase current density of both N (Ca_V_2.2) and L (Ca_V_1.2) Type Ca^2+^ channels when expressed in Xenopus oocytes
Pippucci et al., 2013	Homozygous c.1295delA (p.Asn432fs)	Male		5	Abolished α_2_δ − 2 protein expression in patient
Butler et al., 2018	Compound Heterozygous c.782C > T (p.Pro261Leu) c.3137 T > C (p.Leu1046Pro)	Male		7	Not available
Punetha et al., 2019	Homozygous c.485_486del (p.Tyr162Ter)	Male		7	
	Homozygous c.1778G > C (p.Arg593Pro)	Two affected siblings (1 female, 1 male)		1–2	The present study

*Note*: Summary of the clinical and molecular features of individuals with rare biallelic CACNA2D2 variants (Butler et al., 2018; Edvardson et al., 2013; Pippucci et al., 2013; Punetha et al., 2019).

To overcome this limitation, in this study, we aimed to characterize a previously reported rare homozygous missense variant (p.R593P) found in two siblings with developmental and epileptic encephalopathy (DEE) (Punetha et al., 2019) by studying the effects on the biophysical properties of Ca^2+^ channels, as well as potential consequences on synaptic functions. Our study identified a strongly reduced surface expression and presynaptic localization of mouse α_2_δ‐2 containing the p.R596P mutation, homologous to human p.R593P. Upon heterologous co‐expression, this resulted in altered Ca_V_1.3 current properties, whereas the current density of Ca_V_2.1 was not affected. Nevertheless, homologous expression of p.R596P in hippocampal neurons affected the ability of α_2_δ‐2 to induce a statistically significant increase in the presynaptic abundance of endogenous Ca_V_2.1 and, consequentially, Ca^2+^ transients. Most importantly, our study identified three consequences of the p.R596P mutation on synaptic functions: firstly, reduced, albeit still functional trans‐synaptic coupling to postsynaptic receptors, secondly, reduced presynaptic synapsin clustering in glutamatergic nerve terminals, and thirdly, reduced amplitudes of glutamatergic miniature postsynaptic currents (mEPSCs). Taken together, our data strengthen the hypothesis that the human p.R593P mutation in α_2_δ‐2 is causal for the clinical symptoms of the siblings and demonstrate that disease‐associated α_2_δ mutations can alter channel‐dependent as well as synaptic functions of α_2_δ proteins, both of which may contribute to the pathophysiological mechanism.

## 2 METHODS

### 2.1 Animal and ethical approval

Animal procedures for wild‐type BALB/c mice were performed at the Medical University Innsbruck in compliance with EU and national regulations. Original wild‐type BALB/c mice for starting the breeding colony were bought from Charles River (Germany). The animal facility of the Medical University of Innsbruck was approved as user by the Austrian Federal Ministry of Science, Research and Economy in accordance with §16 TVG 2012, license numbers BMWF‐66.011/0017‐II/3b/2014 and BMWF‐66.011/0067‐II/3b/2014. Mice were maintained in groups of 3–5 per cage at the central animal facility in Innsbruck under standard housing conditions with food and water available ad libitum on a 12 h light/dark cycle. Mice used for breeding were 2 to 14 months old. According to the RRR principle, the number of mice used was kept to the minimum necessary for a statistical representative analysis, which was comparable to numbers reported in previous studies. In total, 13 pregnant mice were used for hippocampal culture preparations. No ethics vote was required, and anonymized information about the patients was quoted from a published study (Punetha et al., 2019).

### 2.2 Cell culture and transfection procedures

#### 2.2.1 Primary cultured hippocampal neurons

Low‐density hippocampal cultures were generated from 16.5‐ to 18‐day‐old embryonic BALB/c mice of either sex as described previously (Geisler et al., 2019; Kaech & Banker, 2006; Obermair et al., 2004). Briefly, pregnant mice were killed by cervical dislocation, embryos were immediately extracted and decapitated. For each culture preparation, 2–8 hippocampi were dissected in cold Hank's balanced salt solution (HBSS, Gibco, cat. no. 14180‐046), pooled, and dissociated by 2.5% trypsin (Gibco, cat. no. 15090‐046) treatment and subsequent trituration. Dissociated neurons were plated at a density of ~3500 cells/cm^2^ (immunolabeling experiments) or 7000 cells/cm^2^ (whole‐cell patch‐clamp recordings), on five 18 mm glass coverslips (Marienfeld Superior, cat. no. 0111580) coated with poly‐l‐lysine (Sigma‐Aldrich, cat. no. P2636) in neuronal plating medium [minimum essential medium (MEM, Gibco, cat. no. 41090‐028), supplemented with 1 mM pyruvic acid (Sigma, cat. no. P2256), 0.6% glucose (Carl Roth, cat. no. HN06.3), and 10% horse serum (Gibco, cat. no. 16050‐122)]. After attachment of neurons for 3–4 h, coverslips were transferred neuron‐side down into a 60 mm culture dish containing a feeder monolayer of glia. Three days after plating, glial proliferation was inhibited with 5 μM Ara‐C (Sigma, cat. no. C6645). Neurons and glia were maintained in NBKO [serum‐free neurobasal medium (Gibco, cat. no. 21103‐049) supplemented with Glutamax (Gibco, cat. no. 35050‐038) and B‐27 (Gibco, cat. no. 17504‐044)] that was changed weekly by replacing one‐third of the volume with fresh maintenance medium. On day in vitro (DIV) six, neurons were transfected with plasmids using Lipofectamine 2000 (Thermo Fisher Scientific, cat. no. 11668019) as described previously (Obermair et al., 2004). 1.5 μg of total DNA was used for co‐transfections at equimolar ratios. For presynaptic Ca^2+^ measurements and whole‐cell patch‐clamp recordings, neurons were used between DIV14 and 17, whereas for immunolabeling experiments, neurons were processed between DIV21 and 25.

#### 2.2.2 tsA201 cells

Human embryonic kidney (HEK)‐293 subclone stably expressing SV40 temperature‐sensitive T antigen (tsA201) cells [ECACC cat. No. 96121229, RRID:CVCL_2737, not listed as a commonly misidentified cell line by the International Cell Line Authentication Committee (ICLAC; http://iclac.org/databases/cross‐contaminations/)] was cultured in Dulbecco's modified Eagle's medium (DMEM; Gibco, cat. no. 11995065) completed with 10% FBS (Gibco, cat. no. 10270106), 0.1 U/mL penicillin, and 0.1 μg/mL streptomycin (PenStrep, Gibco, cat. no. 15140‐122), and were maintained at 37°C in a humidified incubator with 5% CO_2_. Cells were split when they reached ~80% of confluence using 0.5% trypsin–EDTA (Gibco, cat. no. 15400‐054) for detaching adherent cells. The cell's passage number did not exceed 20 passages. For whole‐cell patch‐clamp recordings, tsA201 cells were transiently transfected with α_1_ and β subunit as a control condition or together with WT or mutated α_2_δ‐2 at equimolar ratios using FuGeneHD transfection reagent (Promega, cat. no. E2311) according to the manufacturer protocol. eGFP was always included as a marker for transfected cells. One day after transfection, cells were detached and replated at very low density on poly‐l‐lysine‐coated 35 mm Petri dishes and kept at 30°C and 5% CO_2_ to increase protein expression and inhibit cell proliferation. Cells were used for electrophysiology experiments 48–72 h after transfection. For live‐cell Immunolabeling experiment, cells were plated on 13 mm glass coverslips (Marienfeld Superior, cat. no. 0111530) coated with poly‐l‐lysine. For western blot and live‐cell immunolabeling experiments, tsA201 cells were transfected with 1 μg 2HA‐tagged α_2_δ‐2 together with 0.5 μg eGFP. Experiments were performed 48 hours after transfection.

#### 2.2.3 Lentiviral production

Lentiviruses were produced by transient transfection of confluent Lenti‐X 293 T cells (Takara cat. no. 632180), with the lentiviral expression vectors containing pHR‐βA‐eGFP, pHR‐βA‐eGFP*α_2_δ‐2, or pHR‐βA‐eGFP*α_2_δ‐2_R596P together with psPAX2 (packaging plasmid) and pVSV (envelope plasmid) using Metafectene (Biontex Laboratories, cat. no. T020‐1.0). The following day, sodium butyrate was added (5 mM final concentration) to cells early in the morning to enhance viral production. Six hours later, medium was changed to neuronal plating medium (NPM; consisting of MEM, 10% horse serum, 0.6% glucose, and 1 mM sodium pyruvate), and after 24 h, supernatants containing the viruses were harvested, sterile filtered (0.45 μm syringe filter, Sarstedt, cat. no. 83.1826), aliquoted, and stored at −20°C. Cultured hippocampal neurons were infected immediately after plating with the lentiviral medium supernatant diluted 1:2 in NPM with 3 μg/mL polybrene (Millipore Sigma, cat. no. TR‐1003‐G) and incubated for 4 h in a humidified incubator (5% CO_2_) at 37°C.

### 2.3 Expression vectors and cloning procedures

All plasmids used to transfect primary cultured hippocampal neurons were cloned into an eukaryotic expression plasmid containing a neuronal chicken β‐actin promoter (pβA) to improve neuronal expression. All newly generated constructs were verified by Sanger sequencing (Eurofins Genomics or Microsynth). The cloning procedures to generate the following plasmids were described previously: pβA‐α_2_δ‐2‐v1 (Geisler et al., 2019), pβA‐2HA‐α_2_δ‐2‐v1 (Geisler et al., 2019), pSyn‐GCaMP6f (Brockhaus et al., 2019), pβA‐α_2_δ‐2‐ΔMIDAS (Schopf et al., 2021), pHR‐pβA‐eGFP*α_2_δ‐2‐v1 (Geisler et al., 2019), pβA‐eGFP (Obermair et al., 2004), pHR‐pβA‐mCherry (Geisler et al., 2019), pGFP^−^‐Ca_V_1.3 (Koschak et al., 2001), pCMV‐Ca_V_2.1 (Mullner et al., 2004), β3 (Castellano et al., 1993), and β4 (Etemad et al., 2014).

*pβA‐α*_*2*_ *δ‐2‐v1_R596P*: The p.R596P mutation was introduced by PCR with mutagenesis primers with overlapping extensions. Briefly, α_2_δ‐2‐v1 cDNA sequence was amplified with mutagenesis primers having 21 overlapping bases in two separate PCR reactions using pβA‐α_2_δ‐2‐v1 as template (mouse α_2_δ‐2‐v1 cDNA, GenBank accession number MK327277). For the first PCR reaction, the forward mutagenic primer sequence was 5′‐gaggagatccctcgcagcatgattgacggc‐3′ and the reverse primer sequence was 5′‐gacgacctagactgagctcc‐3′. For the second PCR reaction, the forward primer sequence was 5′‐gacgctgcagagaatttcca‐3′ and the reverse mutagenic primer sequence was 5′‐ catgctgcgagggatctcctccttgttctc‐3′. The two separate PCR products were then used as templates for a final PCR reaction with the two flanking primers to connect the nucleotide sequences. The resulting fragment was then EcoRI/BglII digested and ligated into the corresponding site of pβA‐α_2_δ‐2‐v1 yielding pβA‐α_2_δ‐2‐v1_R596P.

*pβA‐2HA‐α*_*2*_ *δ‐2‐v1_R596P*: pβA‐α_2_δ‐2‐v1_R596P was EcoRI/BglII digested and the fragment containing the p.R596P mutation was ligated into the corresponding site of pβA‐2HA‐α_2_δ‐2‐v1 (mouse α_2_δ‐2‐v1 cDNA sequence with a double hemagglutinin (2HA) tag at the N‐terminus after the predicted signal peptide cleavage site) yielding pβA‐2HA‐α_2_δ‐2‐v1_R596P.

pHR‐pβA‐eGFP*α_*2*_ *δ‐2‐v1_R596P*: pβA‐α_2_δ‐2‐v1_R596P was NheI/RsrII digested and the fragment containing the p.R596P mutation was ligated into the corresponding site of pHR‐pβA‐eGFP*α_2_δ‐2‐v1 yielding pHR‐pβA‐eGFP*α_2_δ‐2‐v1_R596P.

### 2.4 Immunocytochemistry and high‐resolution fluorescence microscopy

Permeabilized or live‐cell immunolabeling of neurons was performed as described previously (Folci et al., 2018; Geisler et al., 2019; Obermair et al., 2004; Schopf et al., 2021; Stanika et al., 2016), and information on primary and secondary antibodies is summarized in Table 2. For permeabilized staining, neurons were fixed with 4% paraformaldehyde and 4% sucrose in PBS (pF) for 20 min at room temperature, washed, and incubated for 30 min in 5% normal goat serum in PBS‐containing 0.2% bovine serum albumin (BSA) and 0.2% Triton X‐100 (PBS/BSA/Triton) to enable membrane permeabilization. Primary antibodies (Table 2) diluted in PBS/BSA/Triton were applied overnight at 4°C and detected by fluorochrome‐conjugated Alexa secondary antibodies incubated for 1 h at room temperature. For live‐cell surface staining of HA‐tagged α_2_δ proteins, transfected neurons were incubated with rat‐anti‐HA antibody diluted in glia‐conditioned neurobasal medium for 10 min at 37°C following quick rinsing in warm HBSS and fixation with pF for 10 min at room temperature. Subsequent washing and blocking steps as well as 1 h incubation with fluorochrome‐conjugated secondary goat anti‐rat Alexa Fluor 594 antibody were conducted with PBS and PBS/BSA, respectively. After washing and fixing cells in pF for 5 min, neurons were permeabilized by blocking solution (PBS/BSA/Triton) and incubated with primary mouse‐anti‐synapsin antibody overnight at 4°C and detected with goat‐anti‐mouse Alexa Fluor 350 antibody. Coverslips were mounted on microscopy slides neuron‐side down in DABCO glycerol solution (Carl Roth, cat. no. 0718.1) to retard photobleaching. Hippocampal neurons were imaged with a BX53 microscope (Olympus) using a 60 X 1.42 numerical aperture (NA) oil immersion objective lens. Fourteen‐bit grayscale images were recorded with a cooled CCD camera (XM10; Olympus) using cellSens Dimension software (Olympus) and further analyzed in MetaMorph software (Molecular Devices). To analyze presynaptic and postsynaptic protein composition, images of randomly selected well‐differentiated and positively transfected cells were acquired with the same exposure and gain settings for all conditions within an experiment. Only cells with medium eGFP expression were selected for the analysis, and overly saturated neurons (based on eGFP levels) were excluded from analysis. Figures were assembled in Adobe Photoshop CS6 using linear adjustments to correct black level and contrast.

### 2.5 Antibodies

Information on primary and secondary antibodies used in this study is summarized in Table 2.

**TABLE 2:** List of antibodies used in this study.

Antibody	Species	Dilution	Source
Anti‐HA	Rat, clone 3F10	1:100 (Live/A594)	Roche (cat. no. 11867423001, RRID:AB_390918)
Anti‐HA	Mouse, clone 5B1D10	1:1000 (WB)	Thermo Fisher Scientific (cat. no. 32–6700, RRID:AB_2533092)
Anti‐GABA_A_R_β2/3_	Mouse, clone bd17	1:500 (A594)	Millipore (cat. no. MAB341, RRID:AB_2109419)
Anti‐synapsin1	Mouse, clone 46.1	1:500 (A350)	Synaptic Systems (cat. no. 106011, RRID:AB_2619772)
Anti‐vGLUT1	Rabbit, polyclonal	1:2000 (A350)	Synaptic Systems (cat. no. 135002, RRID:AB_2315546)
Anti‐Ca_V_2.1	Rabbit, polyclonal	1:2000 (A594)	Synaptic Systems (cat. no. 152203, RRID:AB_2619841)
Anti‐GAPDH	Mouse, clone 6C5	1:10000	Santa Cruz Biotechnology (cat. no. sc‐32 233, PRID:AB_627679)
Alexa Fluor 350	Goat anti‐rabbit	1:500	Thermo Fisher Scientific (cat. no. A‐21068, RRID:AB_2535729)
	Goat anti‐mouse	1:500	Thermo Fisher Scientific (cat. no. A‐21049, RRID:AB_2535717)
Alexa Fluor 594	Goat anti‐rabbit	1:4000	Thermo Fisher Scientific (cat. no. A‐11037, RRID:AB_2534095)
	Goat anti‐mouse	1:4000	Thermo Fisher Scientific (catalog #A‐11032, RRID:AB_2534091)
	Goat anti‐rat	1:4000	Thermo Fisher Scientific (catalog #A‐11007, RRID:AB_10561522)
Secondary antibody coupled to HRP	Goat anti‐mouse IgG [H + L]	1:20 000	Thermo Fisher Scientific (catalog #G21040, RRID:AB_2536527)

*Note*: Summary of primary and secondary antibodies used in immunocytochemistry and western blot.

### 2.6 Image analysis and quantification

#### 2.6.1 Colocalization of synaptic proteins

To visualize the synaptic localization of HA‐tagged α_2_δ subunits, as well as presynaptic (synapsin, vGLUT1, Ca_V_2.1) and postsynaptic proteins (GABA_A_R) of representative images (Figures 6, 7 and 9), lines were manually placed across the single‐/double‐/triple‐labeled synapses and intensities were recorded using the “line scan” function of MetaMorph (Molecular Devices) (Di Biase et al., 2009). Average fluorescence intensities of respective signals were measured along a 3 μm length line, followed by background subtraction, and plotted in Microsoft Excel.

#### 2.6.2 Quantification of fluorescent clusters in single boutons

To analyze the effects of homologous presynaptic expression of WT or mutated α_2_δ‐2 subunit on synapse composition of cultured hippocampal neurons, images from triple‐fluorescence labeling were acquired from the eGFP (green), GABA_A_R_β2/3_ (red), and vGLUT1 (blue) channels. Images were analyzed with a custom‐programmed and semi‐automated MetaMorph journal (Molecular Devices), as described previously (Geisler et al., 2019; Schopf et al., 2021). Briefly, corresponding eGFP and vGLUT1 images were superimposed, eGFP/vGLUT1‐positive varicosities (putative glutamatergic synapses) were randomly chosen as regions of interest (ROIs), and a background region was selected for background subtraction. Axonal varicosities were defined as prominent swellings with higher fluorescence signals compared to the adjacent axonal shaft. Subsequently, GABA_A_R and vGLUT1 grayscale images were measured without thresholding to avoid potential cut‐off of the fluorescent signal. By applying the “shrink region to fit” tool, automatic boundaries were drawn according to the threshold enabling only colocalized clusters to be analyzed, and selected ROIs were then transferred from the eGFP image to the GABA_A_R and vGLUT1 images to measure fluorescent intensities. For the individual synapses in each of the channels, the following parameters were detected in a blinded manner: eGFP threshold area as a measure for bouton size and average and integrated fluorescence intensities providing information on the size and intensity of clusters. In the same manner, the quantification of Ca_V_2.1 together with synapsin signals was performed.

#### 2.6.3 Quantification of live cell surface expression

To analyze presynaptic targeting of HA‐tagged α_2_δ, a slightly modified protocol was used because this staining pattern was not directly co‐localizing with the corresponding presynaptic eGFP signal. Therefore, the presynaptic ROI was dilated by 0.5 μm, in order to avoid false‐positive or false‐negative staining patterns, as described in Schopf et al. (2021). The fluorescent intensity of live‐cell‐stained HA‐tagged α_2_δ in the main three compartments of neurons was determined by measuring the fluorescent intensity along an outlined axon, dendrite, and cell soma.

#### 2.6.4 Quantification analysis

Analyses of all experiments were conducted with Microsoft Excel. For each neuron, an average fluorescence value from minimum of 10 presynaptic varicosities was calculated and plotted in GraphPad Prism 8. For each condition, minimum of 30 cells were considered from three to four independent culture preparations. All values were additionally normalized to the control condition.

### 2.7 Calcium imaging

To examine presynaptic Ca^2+^ influx, GCaMP6f coupled to synaptophysin driven by a synapsin promotor (Brockhaus et al., 2019) was used to conduct Ca^2+^ imaging as described previously (Ablinger et al., 2022). Briefly, primary neurons were transfected at DIV6 with pβA‐SynGCaMP6f and pβA‐mCherry, as a control condition, or together with WT or mutated α_2_δ‐2 subunit. Then, at DIV14‐17, coverslips were mounted in a recording chamber, placed on an inverted microscope (Olympus IX71, 60×, 1.42 NA PlanApo objective), and superfused at 1.0–1.5 mL/min rate with bath solution (32°C), containing (in mM): 145 NaCl, 3 KCl, 1.5 MgCl_2_, 2 CaCl_2_, 11 glucose, and 10 HEPES. To suppress postsynaptic signaling, the following blockers were added to the bath solution (in μM): 10 6‐cyano‐7‐nitroquinoxaline‐2,3‐dione (CNQX, Tocris, cat. no. 1045), 25 DL‐2‐amino‐5‐phosphonovaleric acid (DL‐AP5, Tocris, cat. no. 3693), and 10 bicuculline (Tocris, cat. no. 0109); pH 7.3 adjusted with NaOH. A custom‐built stimulation electrode was positioned with a micromanipulator (MPC‐200, Sutter Instrument) and neurons were stimulated with 50 Hz trains of 1 and 10 current pulses (1 ms, 55 mA) as previously described (Ablinger et al., 2022; Schopf et al., 2021). Ca^2+^ transients were visualized and recorded (20 ms exposure time, frame rate 50 Hz, 200 frames, binning 2, and pixel size: 0.215 μm per pixel) with a CMOS camera (Orca Flash4.0, Hamamatsu), using a halogen lamp light source (HXP 120) in the green channel (excitation: 470/40 nm, emission: 525/50 nm). Recordings were controlled by Micromanager software (Vale Lab, UCSF). As a standard reference, 50 frames were recorded before the stimulus train was triggered and used to calculate the fluorescent baseline and to analyze differences in GCaMP6f presynaptic expression among experimental conditions.

#### 2.7.1 Data analysis

Recordings were analyzed with FIJI/ImageJ software (National Institute of Health) as described previously (Ablinger et al., 2022). Twenty regions of interest (ROIs) per recorded cell were drawn around active presynaptic boutons using the plugin “Time Series Analyzer V3” with an AutoROI diameter of 10 pixels and the “Subtract Background” tool of ImageJ (employing a “rolling ball” algorithm with a radius of 20 pixels ≈ 4.3 μm) was used to remove the background signal. The mean of the four highest fluorescence pixels was calculated for each ROI at each frame by applying a custom‐made macro (Brockhaus et al., 2019) and further analysis was done in Microsoft Excel. To obtain mean sample traces, the changes in fluorescence as ΔF/F0 were calculated for each ROI, and 20 synapses per cell were averaged. The peak fluorescence amplitude for each ROI was obtained by averaging ΔF/F0 values from five frames after the pulse onset. Statistical analysis was performed on the mean peak amplitude of cells in GraphPad Prism 8.

### 2.8 Electrophysiology

#### 2.8.1 tsA201 cells

Ca_V_2.1 and Ca_V_1.3 currents were measured using the whole‐cell patch‐clamp technique in voltage‐clamp mode. Borosilicate glass capillaries (Sutter Instrument, model: BF150‐75‐10) were pulled with a micropipette puller (P‐97, Sutter Instrument) and fire polished using a MF‐830 microforge (Narishige Co). Patch pipettes had a resistance of 2–3.5 MΩ when filled with internal solution containing (in mM): 135 cesium chloride (CsCl, Sigma, cat. no. C3309), 10 Cs‐EGTA (Carl Roth, cat. no. 3054.2), 1 magnesium chloride (MgCl_2_, Sigma, cat. no. M0250), 10 HEPES (Carl Roth, cat. no. 6763.1), and 4 Na_2_ATP (Sigma, cat. no. A1852‐1VL) adjusted to pH 7.3 with 1 M cesium hydroxide (CsOH, Sigma, cat. no. 232068). For measuring Ca^2+^ current of Ca_V_1.3, bath solution with 15 mM Ca^2+^ was used, containing (in mM): 15 calcium chloride (CaCl_2_, Carl Roth, cat. no. 5239.2), 150 Choline‐Cl (Sigma, cat. no. C1879), 1 MgCl_2_, and 10 HEPES, adjusted to pH 7.3 with 1 M CsOH. For measuring Ca^2+^, current through Ca_V_2.1 bath solution with 2 mM Ca^2+^ was used containing (in mM): 2 CaCl_2_, 170 Choline‐Cl, 1 MgCl_2_, 10 HEPES, adjusted to pH 7.3 with 1 M CsOH. Whole‐cell patch‐clamp recordings were performed at room temperature (20–23°C) using an EPC 10 amplifier controlled by Patch Master Software (HEKA Elektronik). Linear leak and capacitive currents were digitally subtracted with a P/4online protocol. Current–voltage (I‐V) relationships were measured by applying 50 ms depolarizing square pulses to various test potentials (from −80 mV to +90 mV) in steps of 5 mV, and holding potential (HP) was set to −80 mV. I‐V curves were fitted according to a Boltzmann equation:

$$I=G_{\mathrm{MAX}}\times \left(V-V_{\mathrm{rev}}\right)/\left(1+e^{-\left(V-V_{0.5}\right)/k}\right)$$

where *I* is the peak current, *G*_MAX_ is the maximum conductance, *V* is the test potential, *V*_rev_ is the extrapolated reversal potential, *V*_0.5_ is the half‐maximal activation voltage, and *k* is the slope factor. The voltage dependence of activation was obtained from the *I*–*V* relationship by calculating the conductance $G=I/\left(V_{\mathrm{rev}}-V\right)$ followed by normalization $\left(G/G_{\mathrm{MAX}}\right)$ and plotting as a function of voltage. The G‐V curve was fitted using the following Boltzmann relationship:

$$G=G_{\mathrm{MAX}}/\left(1+e^{-\left(V-V_{0.5}\right)/k}\right)$$

Series resistance was compensated by 80%–95% and recordings were accepted for analysis when the *I*_peak_ was greater than 100 pA and smaller than 3 nA in size. Voltages were not corrected for the liquid junction potential (−9 mV).

#### 2.8.2 Primary cultured hippocampal neurons

Spontaneous miniature excitatory postsynaptic currents (mEPSCs) of primary cultured hippocampal neurons were recorded at DIV 14 and 15 using the whole‐cell patch‐clamp technique at a holding potential of −70 mV as described previously (Schopf et al., 2021). Patch pipettes were pulled from Borosilicate glass capillaries (Sutter Instrument, model: BF150‐75‐10), fire polished, and had resistances of 2.5–4 MΩ when filled with the following internal solution (in mM): 130 Cs‐methane sulfonate, 10 CsCl, 1 MgCl_2_, 0.1 CaCl_2_, 10 HEPES, 2 EGTA, 4 Mg‐ATP (Sigma, cat. no. G8877), and 0.3 Na‐GTP (Sigma, cat. no. A9187), pH 7.2 with KOH (Carl Roth, cat. no. 6751.1). The bath solution contained the following (in mM): 137 sodium chloride (NaCl, Carl Roth, cat. no. 3957.1), 3 potassium chloride (KCl, Carl Roth, cat. no. 6781.3), 10 HEPES, 2 MgCl_2_, 1.8 CaCl_2_, 10 glucose, 0.05 DL‐AP5, 0.05 bicuculline, and 0.001 tetrodotoxin (TTX, Tocris, cat. no. 1078) pH 7.4 with NaOH. Currents were recorded with an EPC 10 amplifier, controlled by PatchMaster software (HEKA Elektronik Dr. Schulze GmbH, Germany), and analyzed using Mini analysis software (Symaptosoft, USA). Frequency and average amplitude of mEPSC of each cell were normalized to the corresponding mean value of the control condition for each experiment separately.

### 2.9 Western blotting

To determine total protein levels of WT and mutated α_2_δ‐2, whole‐cell lysates (WCL) from tsA201 cells transfected with 2HA‐tagged α_2_δ‐2 were prepared and immunoblotted. At 48 h after transfection, cells were rinsed with phosphate‐buffered saline (PBS) harvested and resuspended in 200 μL ice‐cold RIPA buffer containing: 50 mM Tris–HCl pH 8.0, 150 mM NaCl, 0.1% SDS, 10 mM NaF, 0.5 mM EDTA, 10% Glycerol, and 1% Igepal, supplemented with protease inhibitor cocktail (Thermo Scientific, cat. no. 87785) and lysed on ice for 30 min. Lysates were then cleared by centrifugation at 13 000 × g for 15 min and assayed for total protein concentration using Pierce BCA protein assay kit (Thermo Scientific, cat. no. 23227). Fifty microgram of proteins were then resuspended in NuPAGE™ LDS Sample Buffer (Invitrogen, cat. no. NP0008), with 50 mM dithiothreitol (DTT, Invitrogen, cat. no. NP0004), and separated on precast gradient polyacrylamide gels (NuPAGE™ 4 to 12%, Bis‐Tris, Thermo Scientific, cat. no. NP0336BOX) and transferred to polyvinylidene fluoride (PVDF) membranes with 0.45 μm pore size (Immobilon, cat. no. IPVH00010). Membranes were blocked with 5% milk in Tris‐buffered saline (10 mM Tris‐Base and 0.85% NaCl) with 0.3% Tween (TBS‐T) for 30 min at RT, followed by incubation with the indicated primary antibody overnight at 4°C. The following day membranes were washed three times with TBS‐T and incubated with secondary antibodies coupled to HRP for 60 min and washed three times for 10 min with TBST‐T. The signal was obtained by HRP reaction with SuperSignal™ West Pico PLUS Chemiluminescent Substrate (Thermo Scientific, cat. no. 34580) and membranes were scanned for protein detection with an Intas NEW‐Line ECL ChemoStar Touch Imager HR 9.0 (for HRP) and subsequent protein quantification was performed with Image Studio Light and Microsoft Excel. The signal intensity of 2HA‐α_2_δ‐2 was normalized to the signal intensity of the loading internal control GAPDH. Then, the relative signal intensities were normalized to the corresponding WT 2HA‐α_2_δ‐2 relative signal intensity for each experiment separately.

### 2.10 Homology modeling

The structural homology model of mouse α_2_δ‐2 protein was obtained by superposing the predicted AlphaFold human α_2_δ‐2 (Jumper et al., 2021; Varadi et al., 2022) onto the published cryo‐EM structure of human α_2_δ‐1 subunit within the Ca_V_2.2 complex (PDB code: 7MIY) using PyMOL (The PyMOL Molecular Graphics System, Version 2.3.2. Schrödinger, LLC, New York, NY, USA). PyMOL was used to prepare the figure.

### 2.11 Experimental design and statistical analysis

**FIGURE 1:**
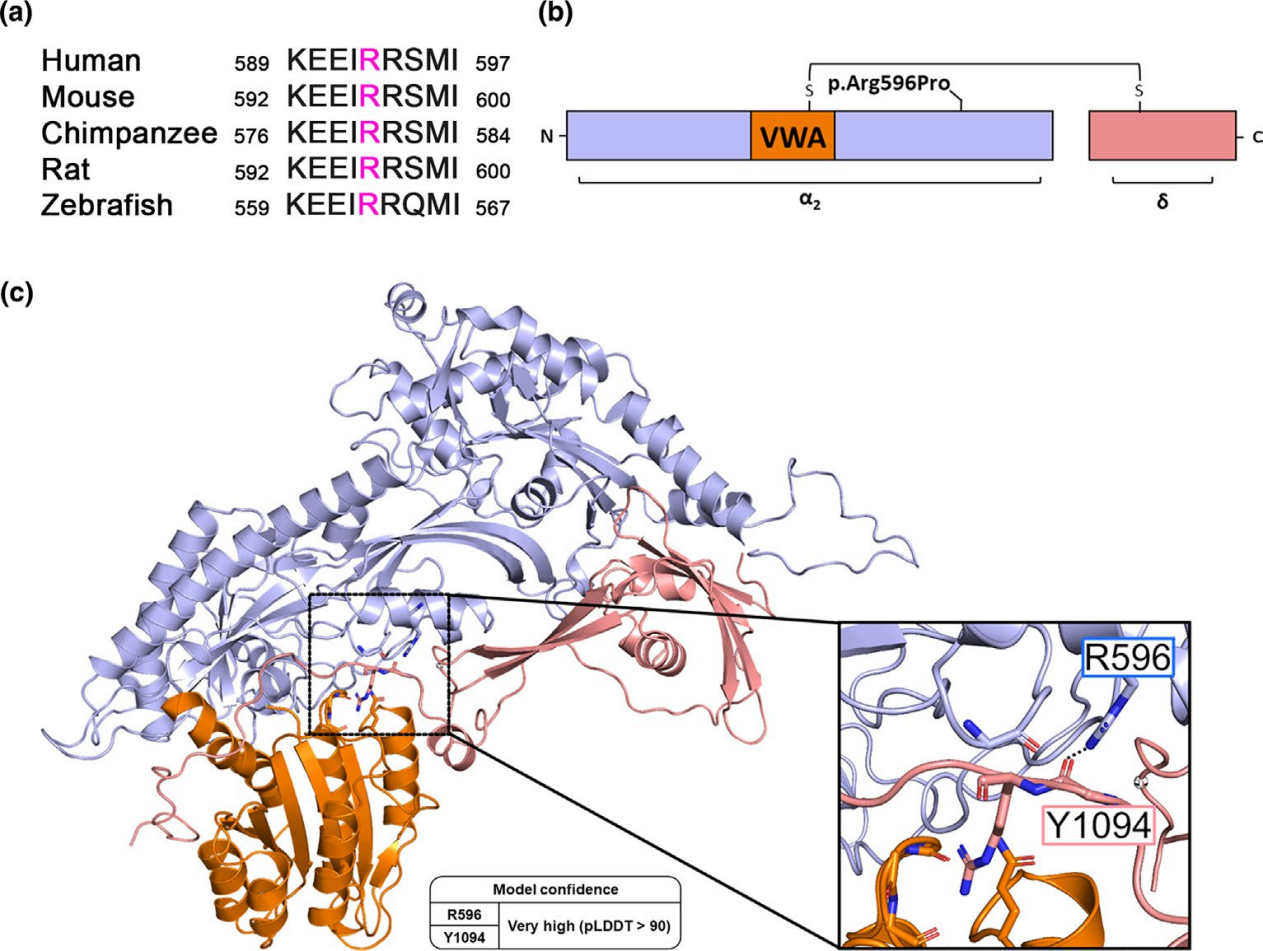
The conserved R596 residue is predicted to have a critical role in stabilizing the interaction between α_2_ and δ peptides. (a) Sequence alignment between α_2_δ‐2 proteins from different species. Position corresponding to human arginine 593 (R596 in mouse α_2_δ‐2) is highlighted in purple. (b) Schematic overview of α_2_δ‐2 protein illustrating the positions of the von Willebrand factor type A domain (VWA) and the p.R596P mutation. (c) Structural homology modeling of mouse α_2_δ‐2 protein based on the published Cryo‐EM structure of the human Ca_V_2.2 channel complex (PDB code: 7MIY) predicts a critical interaction of the side chain of R596 (α_2_ peptide) with the backbone of Y1094 residue (δ peptide) through a hydrogen bond. (Color code: α_2_ peptide in purple, VWA domain in orange, and δ peptide in pink.) AlphaFold per‐residue model confidence scores (pLDDT) for R596 and Y1094 residues are very high (97.38 and 94.25, respectively).

Number of mice used in this study was kept to the minimum necessary for a statistical representative analysis, according to the RRR principle. Where possible, investigators were blinded during experiments and analyses. Three to four independent hippocampal culture preparations were analyzed per experiment and details on cell or bouton numbers are given in the respective figure legends. Electrophysiological recordings in tsA201 cells were obtained from three to four independent experiments (i.e., cell passage and transfection). Details on cell, bouton, or recording numbers are given in the respective figure legends. The distribution of all acquired data was visually assessed using the frequency distribution function of GraphPadPrism 9. In cases where a systematic influence (e.g., tendency to values approaching 0) prevented a normal distribution (e.g., calcium imaging, surface expression analysis in tsA201 cells), statistical analyses were performed on log‐transformed data. All data are shown as mean ± SEM. No test for outliers was conducted and all data points were included in the analysis. Significance levels (*p*‐values) of statistical tests and post hoc analysis are presented in the respective figure legends. The model in Figure 1 was generated with PyMOL (The PyMOL Molecular Graphics System, Version 2.3.2.). Data, graphs, and figures were organized, analyzed, and assembled using MicrosoftExcel, GraphPadPrism 6, SigmaPlot (Systat Software), Adobe PhotoshopCS6, and Affinity Photo. Data contained within the article, and raw data presented in this study are available upon request.

## 3 RESULTS

### 3.1 The evolutionarily conserved arginine 596 (R596) is predicted to be critical for the protein structure of α_2_δ‐2

Amino acid sequence alignment of α_2_δ‐2 from different species shows that human arginine 593 (R593) is an evolutionary conserved amino acid (Figure 1a). Human R593 corresponds to mouse R596 and because we used mouse cDNA in our studies, the residue numbering hereafter refers to mouse α_2_δ‐2. α_2_δ‐2 is post‐translationally cleaved into α_2_ and δ peptides that are linked to each other by disulfide bonds and R596 is located within the α_2_ peptide (Figure 1b). Structural homology modeling predicts an interaction of R596 with tyrosine 1094 (Y1094) within the δ peptide (Figure 1c), suggesting an important role of R596 in maintaining and stabilizing the interaction between the two peptides. Therefore, we hypothesize that R593 is critical for the structural integrity of α_2_δ‐2 and that mutations at this position may alter protein functions.

### 3.2 Reduced membrane expression of epitope‐tagged α_2_δ‐2_R596P upon heterologous expression in tsA201 cells

**FIGURE 2:**
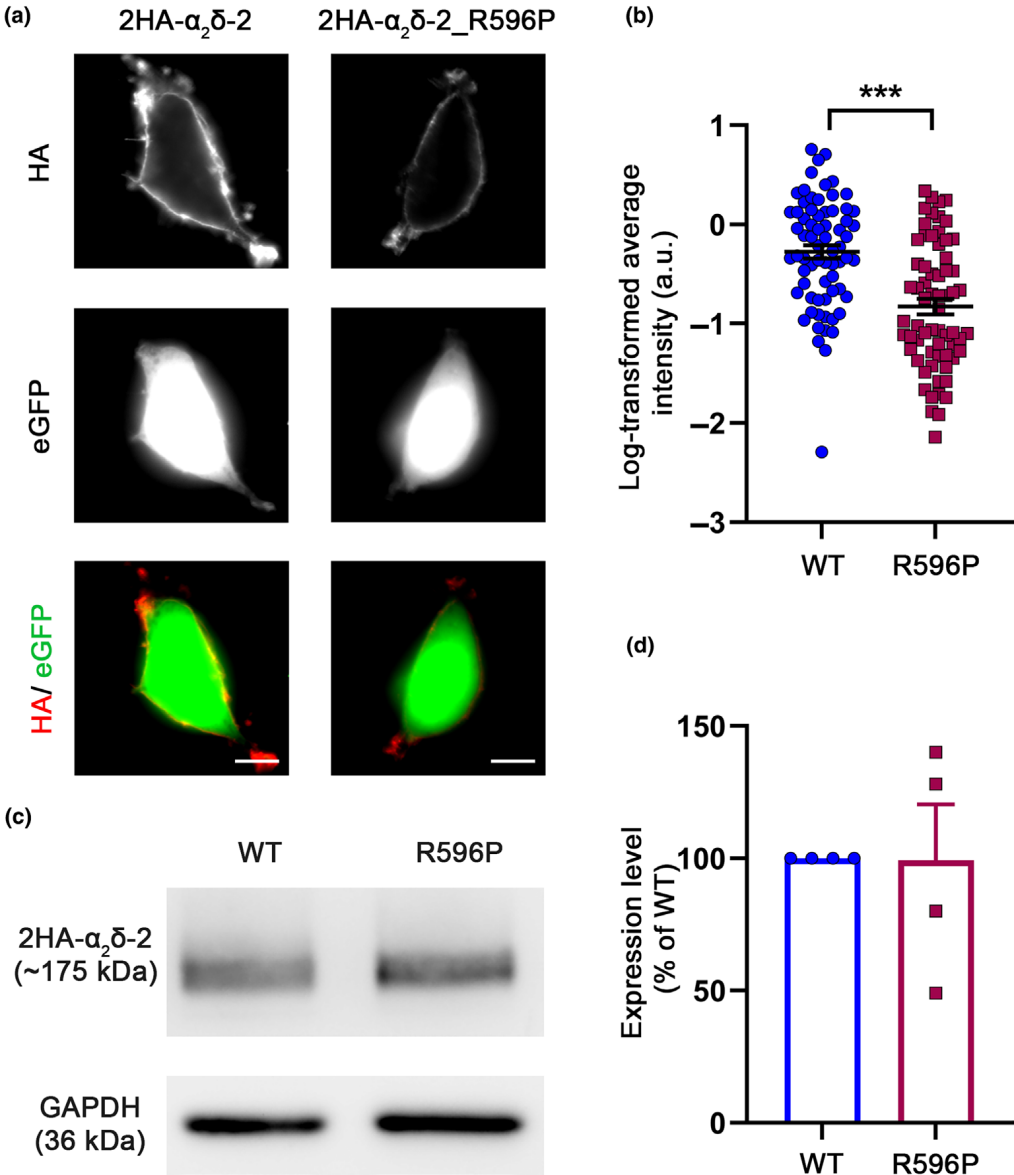
Reduced membrane expression of α_2_δ‐2_R596P compared to WT α_2_δ‐2 in tsA201 cells. tsA201 cells were transfected with soluble eGFP together with either HA‐tagged WT or mutated α_2_δ‐2 (R596P) and were live‐cell labeled with an antibody against the HA epitope. (**a**) Representative images of anti‐HA live‐cell‐labeled tsA201 cells transfected with 2HA‐α_2_δ‐2 or 2HA‐α_2_δ‐2_R596P. Membrane expression of HA‐tagged WT α_2_δ‐2 is identified by a fine‐dotted pattern along the surface of tsA201 cells. In contrast, dots of 2HA‐α_2_δ‐2_R596P labeling are sparsely localized on the cell surface, and overall fluorescence intensity is lower. (**b**) Quantification of α_2_δ‐2 surface expression. Log‐transformed anti‐HA live‐cell‐staining intensities (arbitrary units) are shown for individual cells (dots) and means ± SEM (lines). Data were obtained from three independent experiments and 69 and 66 cells transfected with WT or mutated HA‐tagged α_2_δ‐2 were analyzed, respectively. (**c**) Immunoblot of whole‐cell lysates obtained from tsA201 cells transfected with 2HA‐tagged WT or mutated α_2_δ‐2. α_2_δ‐2 protein was detected with an anti‐HA antibody (upper panel), and anti‐GAPDH labeling was used as loading control (lower panel). (**d**) Quantification of the total protein expression levels of WT and mutated α_2_δ‐2. Relative total protein expression levels of 2HA‐tagged α_2_δ‐2_R596P were normalized to WT 2HA‐tagged α_2_δ‐2 expression levels for each individual experiment (culture preparation). Data were obtained from four independent cell transfections, and values of individual experiments (dots) and mean bars ± SEM (lines) are shown. **Statistics**: (b) unpaired two‐tailed t‐test, *t*_(133)_ = 5.4; ***p < 0.0001; (d) unpaired two‐tailed t‐test performed on raw data, *t*_(6)_ = 0.42; *p* = 0.69. Scale bars, 10 μm.

To study the consequences of the human p.R593P mutation on the protein membrane expression, we introduced the corresponding mutation into the mouse‐coding sequence of α_2_δ‐2 (p.R596P). HA epitope‐tagged wild‐type (WT) or mutated α_2_δ‐2 were expressed together with soluble eGFP in tsA201 cells. Anti‐HA live‐cell labeling of WT α_2_δ‐2 revealed a striking membrane expression identified by a fine‐dotted pattern along the surface of tsA201 cells (Figure 2a, left panel). In comparison to WT α_2_δ‐2, surface expression of the mutated α_2_δ‐2_R596P was strongly reduced (Figure 2a,b). Generally, the p.R596P mutation may impair protein folding resulting in accelerated protein degradation. To test this hypothesis, we performed western blot analysis of whole‐cell lysates from tsA201 cells transfected with WT or mutated HA‐tagged α_2_δ‐2. Overall, α_2_δ‐2 protein levels were comparable between tsA201 cells transfected with WT or mutated proteins, showing that the p.R596P mutation does not alter protein expression levels (Figure 2c,d). Taken together, these data show a strongly reduced membrane expression of HA‐tagged α_2_δ‐2_R596P compared to WT α_2_δ‐2, which is not caused by an altered protein expression level.

### 3.3 The p.R596P mutation alters calcium current properties of the L‐type channel Ca_V_1.3

**FIGURE 3:**
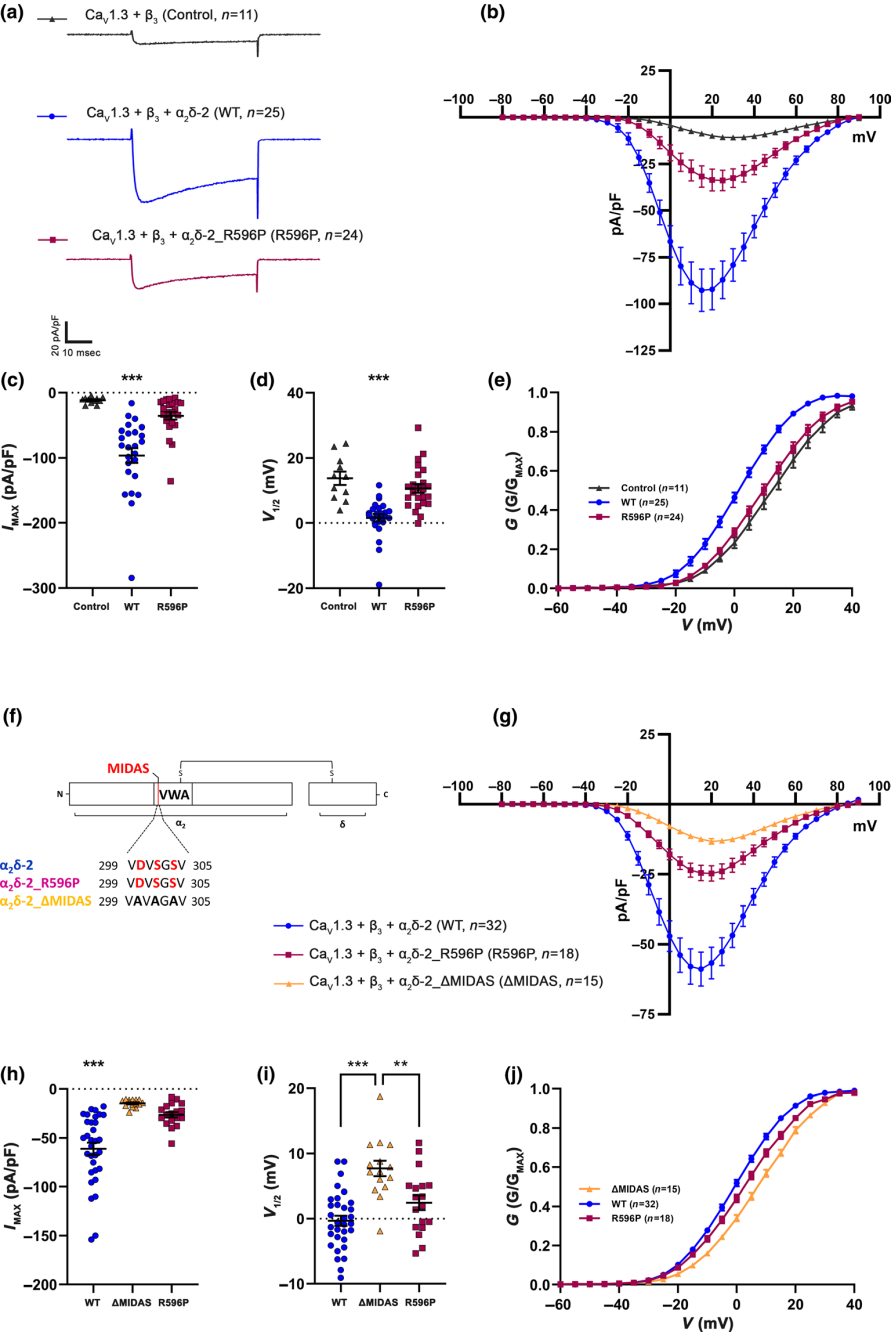
p.R596P reduces current density and alters voltage dependence of activation of the L‐type Ca^2+^ channel Ca_V_1.3. (a–e) Ca^2+^ current properties of Ca_V_1.3 channels recorded from tsA201 cells transfected with Ca_V_1.3 and β_3_ alone (control, gray triangles) or together with wild‐type (WT, blue circles) or mutated α_2_δ‐2 (R596P, pink rectangles). Fifty msec test pulses from a holding potential of −80 mV to +90 mV were applied in 5 mV increments. Representative whole‐cell Ca^2+^ current traces at *V*_MAX_ (a), current–voltage relationships (b), peak current densities (c), and half‐maximal activation potentials (d) of the respective experimental conditions. (e) Fractional activation of the total Ca_V_1.3 channel populations of the respective experimental conditions. **Statistics**: One‐way ANOVA with Tukey's multiple‐comparison test was performed on 11–25 recordings per condition; (c) maximal current density: *F*_(2,57)_ = 21.3; *p* < 0.0001. *P*‐values of the post hoc test for the respective pairwise comparisons: *P* < 0.0001 for control versus WT, *p* = 0.26 for control versus R596P, and *p* < 0.0001 for WT versus R596P. (d) Half‐maximal activation potential: *F*_(2,57)_ = 19.1; *p* < 0.0001. *P*‐values of the post hoc test for the respective pairwise comparisons: *P* < 0.0001 for control versus WT, *p* = 0.38 for control versus R596P, and *p* < 0.0001 for WT versus R596P. Recordings were obtained from three independent experiments. (f) Schematic representation of the different α_2_δ‐2 constructs used in this experiment. The MIDAS motif (highlighted in red) in the α_2_δ‐2_ΔMIDAS construct is mutated to alanines. (g–**j**) Current properties of Ca_V_1.3 channels recorded from tsA201 cells co‐transfected with Ca_V_1.3 and β_3_ together with α_2_δ‐2_ΔMIDAS (ΔMIDAS, orange triangles), wild‐type α_2_δ‐2 (WT, blue circles), and α_2_δ‐2_R596P (R596P, pink rectangles). Current–voltage relationships (g), peak current densities (h), and half‐maximal activation potentials (i) of the respective experimental conditions. (j) Fractional activation of the total Ca_V_1.3 channel population of the respective experimental conditions. **Statistics**: One‐way ANOVA with Tukey's multiple‐comparison test was performed on 15–32 recordings per condition. (h) Maximal current density: *F*_(2,62)_ = 20.1; *p* < 0.0001. *P*‐values of the post hoc test for the respective pairwise comparisons: *P* < 0.0001 for ΔMIDAS versus WT, *p* = 0.40 for ΔMIDAS versus R596P, and *p* < 0.0001 for WT versus R596P. (i) Half‐maximal activation potential: *F*_(2,62)_ = 15.8; *p* < 0.0001. *P*‐values of the post hoc test for the respective pairwise comparisons: *P* < 0.0001 for ΔMIDAS versus WT, *p* = 0.005 for ΔMIDAS versus R596P, and *p* = 0.10 for WT versus R596P. Recordings were obtained from four independent experiments. Significances of post hoc tests between conditions are indicated in the graphs by asterisks (***p < 0.001, **p < 0.01).

Various heterologous co‐expression studies revealed that α_2_δ subunits increase the current density of Ca_V_1 and Ca_V_2 channels when expressed in combination with a β subunit (reviewed in Davies et al., 2007; Dolphin, 2016). Because the single‐channel conductance was not altered by α_2_δ subunits (Barclay et al., 2001; Brodbeck et al., 2002), the proposed mechanism underlying the increase in current density is an increase in the number of functionally expressed channels. In addition to the increase in current density, α_2_δ proteins have been shown to modulate the kinetics and voltage‐dependent properties of Ca^2+^ currents depending on the α_1_ subunit isoform (Obermair et al., 2005; Tuluc et al., 2007). Hence, we next analyzed whether and how the p.R596P mutant with its strongly reduced membrane expression affects the channel‐dependent functions of α_2_δ‐2. Because α_2_δ‐2 is predominantly expressed in cerebellar Purkinje cells and inner hair cells of the cochlea, we focused on the most likely Ca_V_ subunit partners of α_2_δ‐2 in these cells, Ca_V_2.1 (cerebellum) and Ca_V_1.3 (inner hair cells) (Fell et al., 2016; Schlick et al., 2010). Moreover, these two Ca_V_ channel isoforms have a distinct subcellular distribution in neurons of the CNS. While Ca_V_1.3 channels are predominantly localized at postsynaptic sites (Stanika et al., 2016), Ca_V_2.1 channels are located at presynaptic terminals (Etemad et al., 2014). Hence, studying the consequences of the p.R596P mutation on these Ca_V_ channel isoforms provides insight into the involvement of pre‐ and postsynaptic compartments in the pathophysiology of p.R596P variant. We first studied the current properties of the L‐type channel Ca_V_1.3 by performing whole‐cell patch‐clamp recordings in tsA201 cells transfected with Ca_V_1.3 and β_3_ without (control) or together with WT or mutated α_2_δ‐2. As previously reported, the presence of α_2_δ‐2 significantly increased Ca_V_1.3 current amplitudes and left shifted the voltage dependence of activation (Figure 3a–e). More importantly, co‐expression of α_2_δ‐2_R596P failed to increase the current density of Ca_V_1.3 to WT levels and prevented the left shift in the current–voltage relationship (Figure 3d; Table 3).

**TABLE 3:** Current properties of Ca_V_1.3 in tsA201 cells.

	Ca_V_1.3/β_3_			Ca_V_1.3/β_3_		
	Control	WT	R596P	ΔMIDAS	WT	R596P
Current density (pA/pF)	−11.7 ± 1.5	−96.2 ± 11.3	−35.5 ± 5.8	−14.3 ± 1.0	−61.0 ± 6.3	−26.2 ± 2.7
*V*_1/2_ (mV)	13.7 ± 2.0	1.5 ± 1.1	10.6 ± 1.3	7.7 ± 1.1	−0.3 ± 0.7	2.4 ± 1.1
*V*_rev_ (mV)	80.0 ± 1.4	70.3 ± 0.8	75.8 ± 1.0	71.1 ± 1.5	66.7 ± 0.6	68.2 ± 0.8
*n*	11	25	24	15	32	18

*Note*: All values are presented as mean ± SEM and were obtained from >3 independent experiments. *V*_1/2_ and *V*_rev_ parameters were obtained by fitting the *I*–*V* curves to a Boltzmann function. *V*_1/2_, Half‐maximal activation potential; *V*_rev_, extrapolated reversal potential; and *n*, number of recordings. For statistics, see Figure 3.

In theory, two mechanisms can contribute to the observed reduction in current density obtained when α_2_δ‐2_R596P was co‐expressed with Ca_V_1.3. Firstly, the p.R596P mutant may fail to support membrane trafficking of the channel complex. Secondly, the right shift in the current–voltage relationship may reduce the driving force at more positive potential, which is a known mechanism of α_2_δ protein action on L‐type calcium channels (Obermair et al., 2008; Tuluc et al., 2007). Similar half‐maximal activation potential obtained for cells expressing α_2_δ‐2_R596P or no α_2_δ (control) supports a failure in membrane trafficking. Hence, to test if Ca_V_1.3 channels are still partly modulated by α_2_δ‐2_R596P, we compared the current properties with Ca_V_1.3 channels co‐expressed with an α_2_δ‐2_ΔMIDAS (DxSxS motif is mutated to alanines) deletion construct. The metal ion‐dependent adhesion site (MIDAS) within the VWA domain of α_2_δ‐1 and α_2_δ‐2 has been previously shown to play a key role in channel trafficking. More precisely, mutating the MIDAS motif results in ER retention of α_2_δ proteins and thereby completely abolishes their ability to enhance membrane expression (Canti et al., 2005; Hoppa et al., 2012). Mean current density of channels co‐expressed with p.R596P was not significantly different from that of channels co‐expressed with α_2_δ‐2_ΔMIDAS (Figure 3h; Table 3). However, there was a statistically significant difference in the half‐maximal activation potential (Figure 3i; Table 3). Moreover, in 62% of cells co‐transfected with α_2_δ‐2_ΔMIDAS, no current could be detected. In contrast, only 35% and 13% of cells co‐transfected with p.R596P and WT α_2_δ‐2, respectively, showed no current [n numbers: α_2_δ‐2_ΔMIDAS (45), α_2_δ‐2_ R596P (40), and WT (53)]. Together, these data suggest that in contrast to α_2_δ‐2_ΔMIDAS, α_2_δ‐2_R596P proteins, despite showing a strongly reduced membrane expression, can still interact and modulate Ca_V_1.3 channels at a very basic level.

### 3.4 The p.R596P mutation does not compromise current properties of the P/Q‐type channel Ca_V_2.1

**FIGURE 4:**
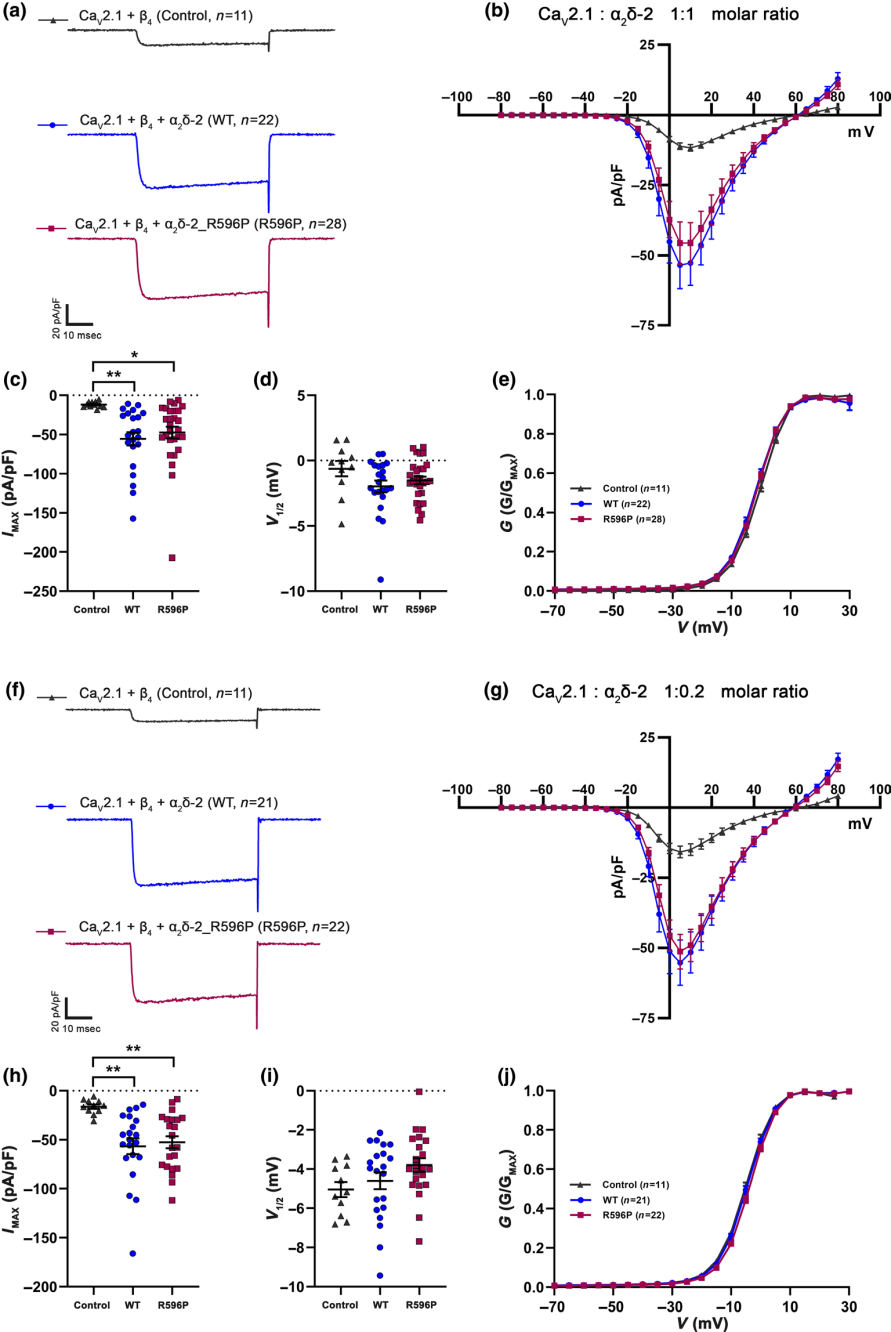
The p.R596P mutation does not compromise the current properties of the P/Q‐type channel Ca_V_2.1. (a–j) Current properties of Ca_V_2.1 recorded from tsA201 cells transfected with Ca_V_2.1 and β_4_ alone as a control (control, gray triangles), together with WT (WT, blue circles), or mutated α_2_δ‐2 (R596P, pink rectangles). (a–e) Recordings obtained from tsA201 cells transfected with the three subunits Ca_V_2.1/β_4_/α_2_δ‐2 in a molar ratio of 1:1:1, while (f–j) are recordings obtained from tsA201 cells transfected with the three subunits in a molar ratio of 1:1:0.2. Fifty msec test pulses from a holding potential of −80 mV to +80 mV were applied in 5 mV increments. (a, f) Representative whole‐cell Ca^2+^ current traces at V_MAX_. Current–voltage relationships (b, g), mean peak current densities (c, h), and mean half‐maximal activation potentials (d, i). **Statistics**: (c, d) One‐way ANOVA with Tukey's multiple‐comparison test was performed on 11–28 recordings per condition obtained from three independent experiments. (c) Maximal current density, *F*_(2,58)_ = 5.5; *p* = 0.0067. *P*‐values of the post hoc test for the respective comparisons: *P* = 0.01 for control versus WT, *p* = 0.02 for control versus R596P, and *p* = 0.72 for WT versus R596P. (d) Half‐maximal activation potential, *F*_(2,58)_ = 2.0; *p* = 0.15. *P*‐values of the post hoc test for the respective comparisons: *P* = 0.12 for control versus WT, *p* = 0.37 for control versus R596P, and *p* = 0.65 for WT versus R596P. (h, i) One‐way ANOVA with Tukey's multiple‐comparison test was performed on 11–22 recordings per condition obtained from three independent experiments. (h) Maximal current density, *F*_(2,51)_ = 7.4; *p* = 0.0016. *P*‐values of the post hoc test for the respective comparisons: *P* = 0.002 for control versus WT, *p* = 0.005 for control versus R596P, and *p* = 0.89 for WT versus R596P. (i) Half‐maximal activation potential, *F*_(2,51)_ = 2.2; *p* = 0.117. P‐values of the post hoc test for the respective comparisons: *P* = 0.76 for control versus WT, *p* = 0.13 for control versus R596P, and *p* = 0.29 for WT versus R596P. Significances of post hoc tests between conditions are indicated in the graphs by asterisks (**p < 0.01, *p = 0.02).

We next tested the consequences of the p.R596P mutation on the biophysical properties of Ca^2+^ currents mediated by the P/Q‐type channel Ca_V_2.1. Compared to the control condition without α_2_δ‐2, co‐expression of Ca_V_2.1 and β_4_ together with the p.R596P mutant resulted in a fivefold increased current density. This increase was basically indistinguishable from the positive control group (co‐expression of WT α_2_δ‐2, Figure 4a–e; Table 4), suggesting that Ca_V_2.1 function is not compromised by the mutation. However, because Ca_V_2.1 channels showed an upper limit of membrane expression in superior cervical ganglion neurons (Scott & Kammermeier, 2017), small changes in current densities may be masked. This prompted us to repeat the analysis of P/Q‐type currents in tsA201 cells transfected with a molar ratio of 1:0.2 (α_1_:α_2_δ‐2) instead of 1:1. However, also the low amount of α_2_δ‐2 cDNA used in these transfections neither resulted in a reduced Ca_V_2.1 current density upon co‐expression of WT α_2_δ‐2, or upon co‐expression of mutated α_2_δ‐2 (Figure 4f–j; Table 4). Together this shows that α_2_δ‐2_R596P, despite showing an extremely low membrane expression, is still able to increase the functional membrane expression of Ca_V_2.1 channels.

**TABLE 4:** Current properties of Ca_V_2.1 in tsA201 cells.

	Ca_V_ 2.1/β_4_/α_2_δ‐2 (1:1:1 molar ratio)			Ca_V_ 2.1/β_4_/α_2_δ‐2 (1:1:0.2 molar ratio)		
	Control	WT	R596P	Control	WT	R596P
Current density (pA/pF)	−11.9 ± 1.1	−55.5 ± 8.5	−47.4 ± 7.6	−16.3 ± 2.2	−56.6 ± 8.0	−52.5 ± 6.1
*V*_1/2_ (mV)	−0.6 ± 0.5	−1.9 ± 0.4	−1.5 ± 0.2	−5.0 ± 0.3	−4.5 ± 0.4	−3.8 ± 0.3
*V*_rev_ (mV)	47.0 ± 0.5	44.7 ± 0.3	46.0 ± 0.3	44.4 ± 1.0	44.8 ± 0.4	45.7 ± 0.4
*n*	11	22	28	11	21	22

*Note*: All values are presented as mean ± SEM and were obtained from three independent experiments. *V*_1/2_ and *V*_rev_ parameters were obtained from fitting the *I*–*V* curves to a Boltzmann function. *V*_1/2_, half maximal activation potential; *V*_rev_, extrapolated reversal potential; *n*, number of recordings. For statistics see Figure 4.

### 3.5 Strongly reduced neuronal surface expression and presynaptic targeting of epitope‐tagged α_2_δ‐2_R596P

**FIGURE 5:**
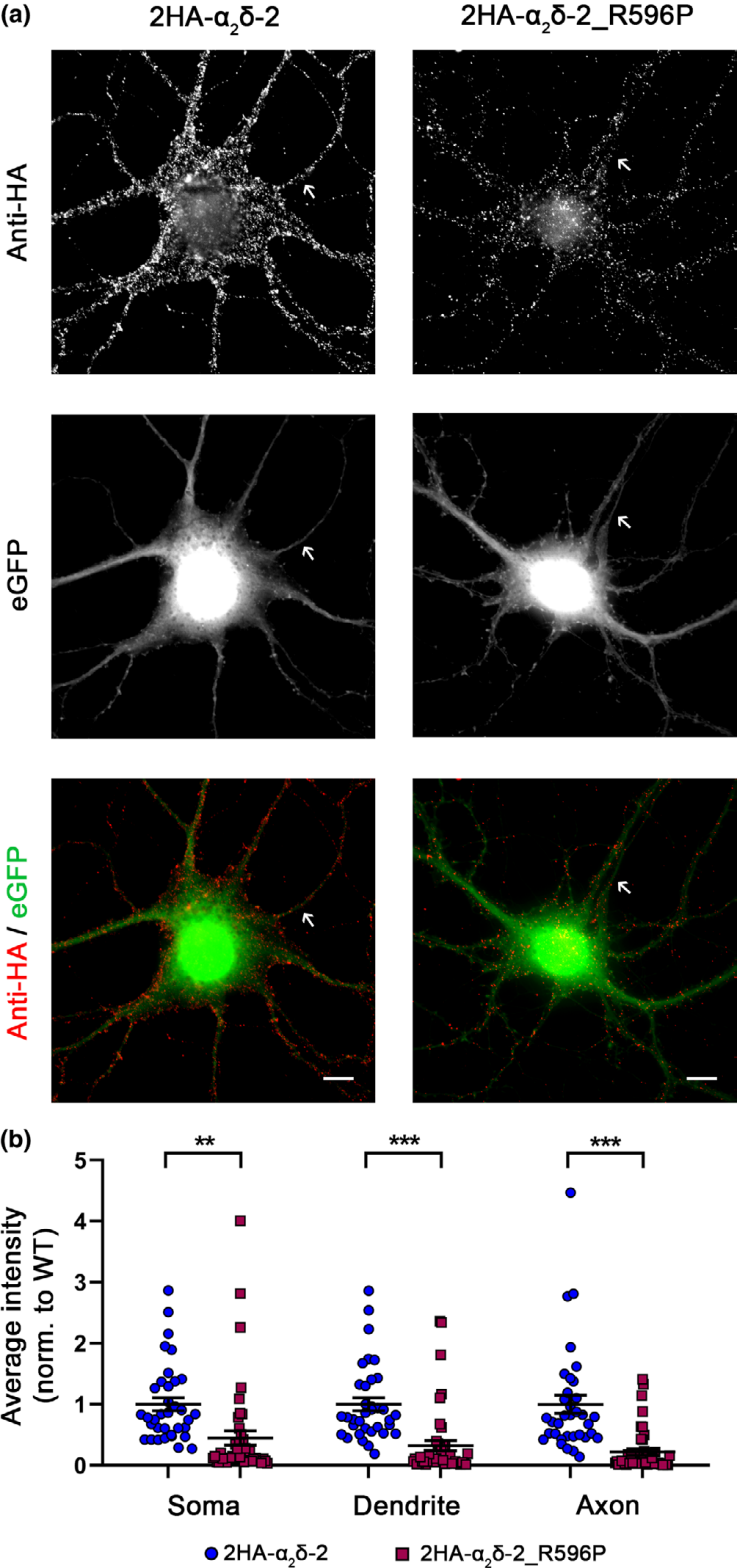
Strongly reduced membrane expression of α_2_δ‐2_R596P in differentiated cultured hippocampal neurons. (a) Representative examples of primary cultured hippocampal neurons transfected with soluble eGFP together with either HA‐tagged WT (2HA‐α_2_δ‐2) or mutated (2HA‐α_2_δ‐2_R596P) α_2_δ‐2. Anti‐HA live‐cell labeling demonstrates a reduced staining intensity of α_2_δ‐2_R596P in the soma, dendrites, and the axon (indicated by an arrow) compared to WT α_2_δ‐2. (b) Quantification of the average HA fluorescent intensities in the three compartments shows that the surface expression of mutated α_2_δ‐2 is strongly reduced compared to WT α_2_δ‐2. Graphs show values for individual cells (dots) and mean ± SEM (lines). All values were normalized to the mean of the WT 2HA‐α_2_δ‐2 fluorescence intensity within each culture preparation. Data were obtained from three independent culture preparations; 35 and 45 cells expressing WT or mutated HA‐tagged α_2_δ‐2 were analyzed, respectively. **Statistics**: Unpaired *t*‐test, soma: *T*_(78)_ = 3.4; **p = 0.0011, dendrite: *T*_(78)_ = 5.1; ***p < 0.0001, axon: *T*_(78)_ = 5.5; ***p < 0.0001. Scale bars, 10 μm.

So far, we studied the functional consequences of the p.R596P mutation in α_2_δ‐2 on the membrane expression and Ca^2+^‐channel properties upon heterologous expression in tsA201 cells. While this expression system is ideally suited to study the consequences of the mutated α_2_δ‐2 protein on distinct α_1_ subunits and in the absence of endogenously expressed auxiliary subunits, it is essential to investigate the behavior and the consequences of the mutated protein in its native environment, namely neurons of the CNS. Therefore, we homologously expressed HA epitope‐tagged WT or mutated α_2_δ‐2 together with soluble eGFP in mouse cultured hippocampal neurons. Live‐cell immunostaining revealed that, similar to heterologous expression, α_2_δ–2_R596P showed a strongly reduced surface expression compared to WT α_2_δ‐2, in all three main neuronal compartments, the soma, dendrites, and axons (Figure 5a).

**FIGURE 6:**
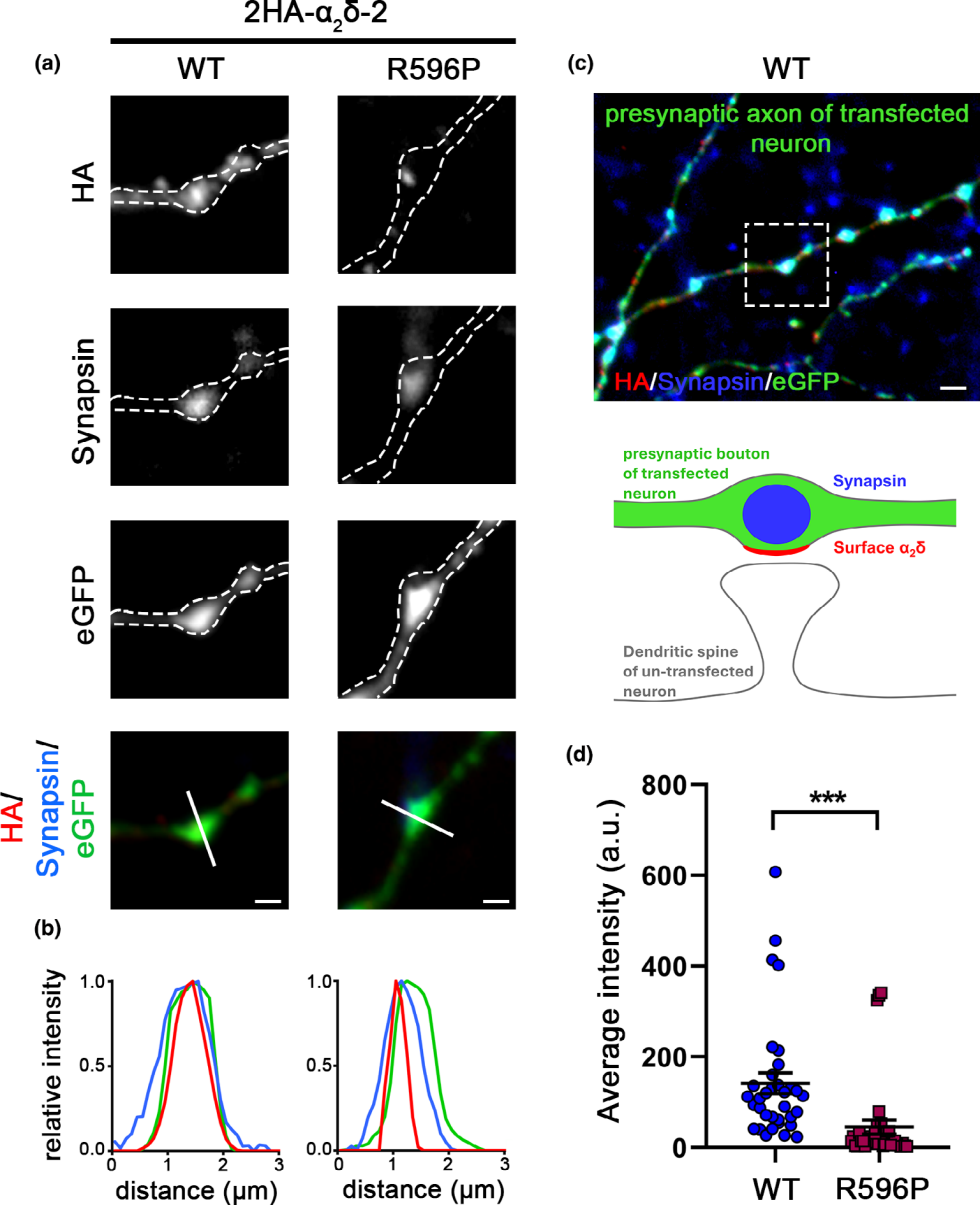
Reduced presynaptic targeting of α_2_δ‐2_R596P. (a) Representative presynaptic boutons of transfected cultured hippocampal neurons, identified by the eGFP expression and synapsin clustering, are outlined by a dashed line. Live‐cell staining shows that α_2_δ‐2_R596P is expressed at the surface of presynaptic boutons. Scale bars, 1 μm. (b) Line scan analysis of 2HA‐α_2_δ‐2 (red), synapsin (blue), and eGFP (green) staining pattern of representative images supports a presynaptic localization by the overlapping peaks of HA‐label (red) in relation to synapsin (blue) and eGFP (green). (c) The surrounding region of the selected WT synapse in (a) and a sketch summarizing the observed labeling pattern. Scale bar, 2 μm. (d) Average fluorescence intensity measurements of the HA signal in positively transfected boutons revealed a strong reduction in presynaptic localization of α_2_δ‐2_R596P compared to WT α_2_δ‐2. **Statistics**: Graph shows mean values of minimally five synapses for individual cells (dots) and means ± SEM (lines). Data were obtained from three independent culture preparations and 34 and 35 cells expressing WT or mutated HA‐tagged α_2_δ‐2 were analyzed, respectively. Statistics: unpaired two‐tailed *t*‐test, *t*_(67)_ = 3.0; ***p = 0.0037.

As α_2_δ‐2 is particularly expressed in presynaptic terminals (Geisler et al., 2019), we next analyzed the presynaptic localization of α_2_δ‐2_R596P. To this end, presynaptic boutons were identified by the clustering of synapsin protein within axonal varicosities as visualized by the eGFP fluorescence. Analysis of presynaptic boutons expressing HA‐tagged α_2_δ‐2 proteins revealed a presynaptic localization of α_2_δ‐2_R596P, similar to WT α_2_δ‐2 (Figure 6a), as indicated by the colocalization and the line scan analysis of the 2HA‐α_2_δ‐2 (red), synapsin (blue), and eGFP (green) staining pattern of representative images (Figure 6b). However, quantitative analysis revealed that, compared to WT α_2_δ‐2, overall presynaptic targeting of α_2_δ‐2_R596P was strongly reduced (Figure 6d).

### 3.6 Homologous expression of WT or R596P‐mutated α_2_δ‐2 differentially affects presynaptic differentiation

**FIGURE 7:**
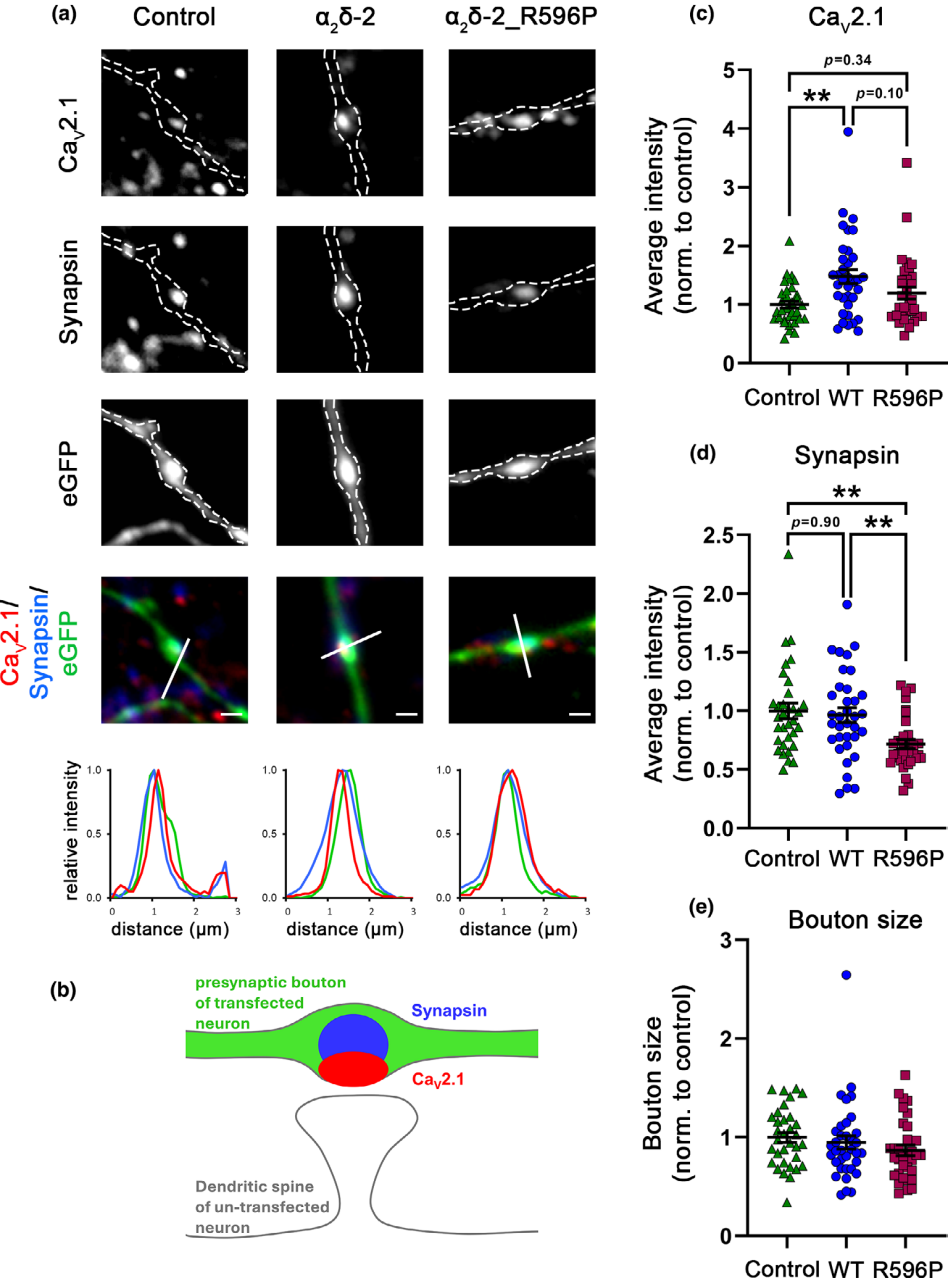
Homologous expression of α_2_δ‐2_R596P in hippocampal neurons fails to induce a statistically significant increase in presynaptic Ca_V_2.1 clustering and decreases presynaptic synapsin abundance. (a) Representative micrographs of presynaptic boutons of cultured hippocampal neurons transfected with eGFP alone (control) or co‐transfected together with either WT (α_2_δ‐2) or mutated (α_2_δ‐2_R596P) α_2_δ‐2. Immunofluorescent signals of Ca_V_2.1 channels colocalize with presynaptic synapsin clusters (see also co‐localizing peaks of fluorescence signals in line scan analysis). (b) Sketch depicting the expected staining pattern in (a). (c) Quantification of presynaptic Ca_V_2.1 cluster intensity shows that contrary to WT, presynaptic expression of the p.R596P mutant fails to induce a significant increase in presynaptic Ca_V_2.1 clustering. (d) Quantification of presynaptic synapsin abundance shows a strong reduction in synaptic synapsin intensity upon expression of the p.R596P mutant. (e) Quantification of boutons size, as identified by the eGFP fluorescence area, shows no difference between the different experimental conditions. **Statistics**: Graphs of Ca_V_2.1 and synapsin average intensities and bouton size show values for individual cells (dots) and means ± SEM (lines). Cells were obtained from three independent culture preparations. One‐way ANOVA with Tukey's multiple‐comparison test. A total of 33–35 cells per condition, (c) *F*_(2,98)_ = 6.1, **p = 0.0031. *P*‐values of the post hoc test for the respective pairwise comparisons: *P* = 0.002 for control versus WT, *p* = 0.34 for control versus R596P, and *p* = 0.12 for WT versus R596P. (d) *F*_(2,98)_ = 7.0, **p = 0.0013. *P*‐values of the post hoc test for the respective pairwise comparisons: *P* = 0.89 for control versus WT, p = 0.002 for control versus R596P, and *p* = 0.01 for WT versus R596P. (e) *F*_(2,98)_ = 1.2, *p* = 0.29. *P*‐values of the post hoc test for the respective pairwise comparisons: *P* = 0.81 for control versus WT, *p* = 0.27 for control versus R596P, and *p* = 0.61 for WT versus R596P. Significances of post hoc tests between conditions are indicated in the graphs by asterisks (**p < 0.01). Scale bar, 1 μm.

The unaltered Ca_V_2.1 current observed when p.R596P was co‐expressed with the channel in tsA201 cells (Figure 4) suggests that despite the strong reduction in membrane expression, the p.R596P mutant was still able to traffic Ca_V_2.1 channels to the plasma membrane. However, and as discussed above, Ca_V_2.1 channel membrane expression ceiling may obscure small differences in channel modulation. It has previously been shown that homologous expression of α_2_δ‐1 in cultured neurons increases synaptic Ca_V_2.1 channel clustering (Ablinger et al., 2022; Hoppa et al., 2012). Here, we show that WT α_2_δ‐2 similarly induces an increase in endogenous Ca_V_2.1 clustering at presynaptic terminals compared to control neurons expressing eGFP alone (Figure 7a,c). This observation allowed us to test, whether this property of α_2_δ‐2 is compromised by the mutation. In contrast to WT α_2_δ‐2, α_2_δ‐2_R596P expression resulted only in a slight but not significant increase in presynaptic Ca_V_2.1 expression (Figure 7c), which is neither different from control (eGFP alone) nor from WT α_2_δ‐2 (WT). Surprisingly, however, homologous expression of α_2_δ‐2_R596P significantly reduced presynaptic synapsin clustering, which was not observed in the control groups (eGFP or WT α_2_δ‐2; Figure 7d). The reduction in synapsin expression was not the consequence of an altered bouton size, as evidenced by quantitative analysis of the size of eGFP‐positive varicosities (Figure 7e). Moreover, the decrease in presynaptic synapsin clustering suggests that the p.R596P mutant, despite its low membrane expression, competes with endogenously expressed α_2_δ proteins (α_2_δ‐1 to ‐3), thereby mediating a dominant‐negative effect resulting in defective presynaptic differentiation.

### 3.7 The p.R596P mutation affects the ability of α_2_δ‐2 to increase presynaptic calcium transients

**FIGURE 8:**
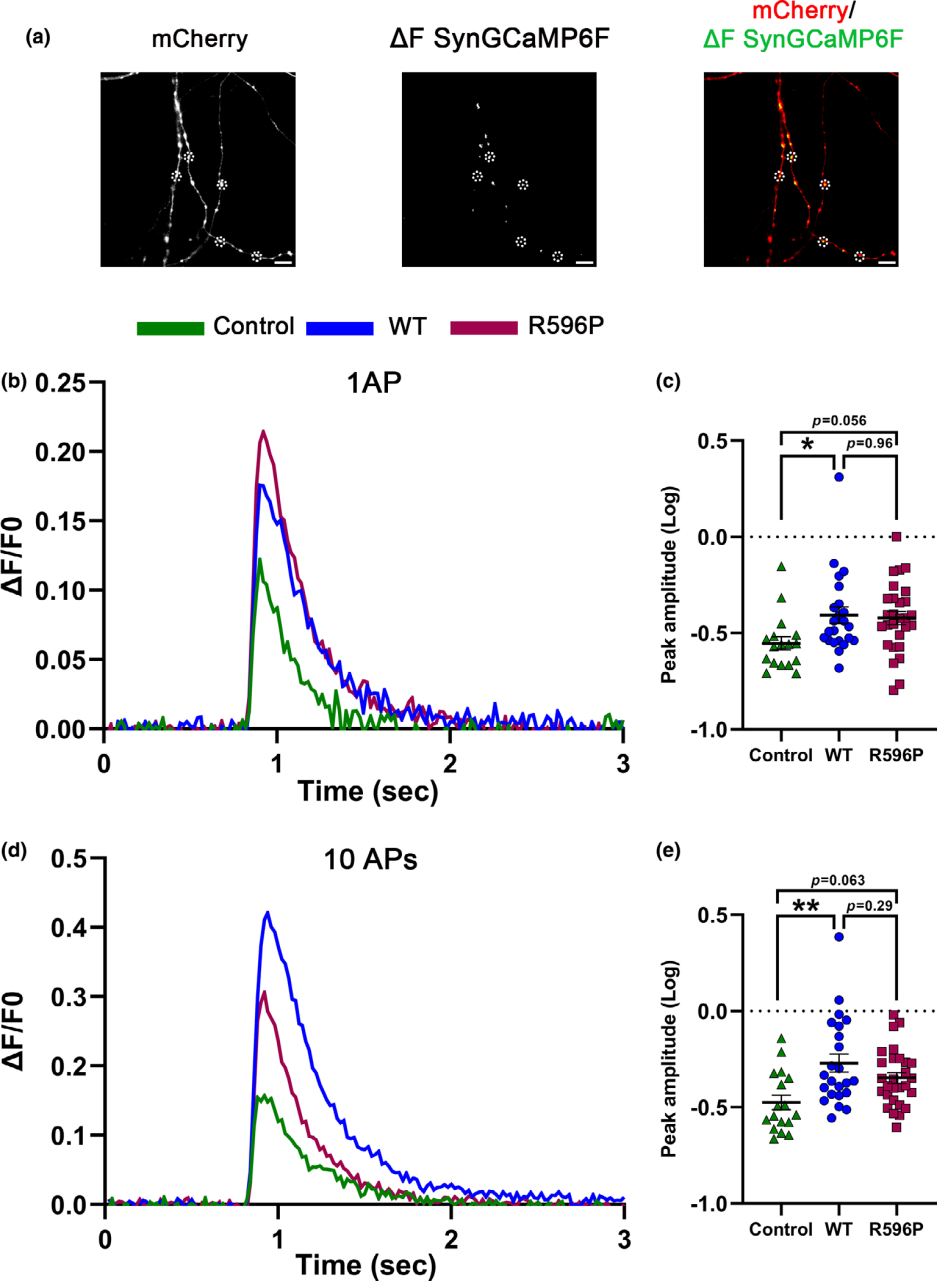
Presynaptic Ca^2+^ transients of neurons expressing WT α_2_δ‐2 and α_2_δ‐2_R596P in comparison with control neurons. (a) Fluorescent micrographs of representative presynaptic varicosities identified by the mCherry fluorescence. The presynaptic Ca^2+^ signal (ΔSynGCaMP6f) was calculated by subtracting the control SynGCaMP6f fluorescence at baseline from the SynGCaMP6f fluorescence at the maximal response. Scale bar 5 μm. Mean fluorescence traces (b, d), and quantification of the peak fluorescence (c, e) of presynaptic Ca^2+^ signals (ΔF/F of SynGCaMP6f) elicited by 1 action potential (AP), or 10 APs at 50 Hz stimulations, in cultured hippocampal neurons co‐transfected with SynGCaMP6f and mCherry alone (control, green, triangles) or together with either WT (WT, blue, circles) or mutated α_2_δ‐2 (R596P, pink, rectangles). (b, d) Lines show the mean fluorescence traces from 17 to 28 cells per condition from four independent culture preparations. Quantification of peak fluorescent amplitudes (log) in response to stimulations with 1 (c) or 10 APs (e) shows values for individual cells (symbols) and means ± SEM (lines). Each symbol represents the mean peak fluorescence signal of 20 synapses measured from one neuron. **Statistics**: One‐way ANOVA with Tukey's multiple‐comparison test: 1 AP: *F*_(2,65)_ = 3.6, *p* = 0.038. *P*‐values of the post hoc test for the respective pairwise comparisons: *P* = 0.03 for control versus WT, *p* = 0.06 for control versus R596P, and *p* = 0.96 for WT versus R596P. 10 AP: *F*_(2,65)_ = 6.2, *p* = 0.003. *P*‐values of the post hoc test for the respective pairwise comparisons: *P* = 0.002 for control versus WT, *p* = 0.06 for control versus R596P, and *p* = 0.28 for WT versus R596P. Significances of post hoc tests between conditions are indicated in the graphs by asterisks (**p < 0.01, *p = 0.038).

Homologous expression of α_2_δ‐2_R596P fails to induce a significant increase in presynaptic Ca_V_2.1 channel abundance. This is in contrast to the unaltered biophysical current properties (confer Figure 4) and hence suggests a mild disruption in the interaction of α_2_δ‐2 with Ca_V_2.1. Moreover, the decrease in presynaptic synapsin clustering is a sign of defective presynaptic differentiation, which, in theory, can also affect the expression of other presynaptic Ca_V_ channel isoforms. Therefore, we next tested whether and to what extent presynaptic Ca^2+^ transients were affected by WT or R596P α_2_δ‐2. To this end, cultured hippocampal neurons were transfected with the genetically encoded GCaMP6f Ca^2+^ indicator coupled to synaptophysin [SynGCaMPF6f; Brockhaus et al., 2019)], together with soluble mCherry (Figure 8a). Comparable synaptic expression of GCaMP6f across conditions was confirmed by analyzing the fluorescent signal intensity in synaptic boutons at baseline (control: 40.37 ± 2.59, WT: 35.31 ± 0.86, R596P: 38.35 ± 1.54; one‐way ANOVA with Tukey's multiple‐comparison test: *F*_(2,65)_ = 2.14, *p* = 0.13). Consistent with previous findings, WT α_2_δ‐2 increased presynaptic peak Ca^2+^ amplitudes in response to 1 and 10 action potentials (AP) triggered by field stimulation at a frequency of 50 Hz (Figure 8b–e). This was evident when plotting the mean traces (Figure 8b,d) and by analyzing the peak Ca^2+^ signals of all individual cells (Figure 8c,e). Surprisingly, the p.R596P mutant also increased presynaptic peak Ca^2+^ amplitudes similar to WT α_2_δ‐2 in response to 1 AP (Figure 8b,c). However, in response to 10 Aps, the mean peak Ca^2+^ amplitude of the p.R596P condition is lower, albeit not significantly different, compared to the WT α_2_δ‐2 condition. Moreover, and in contrast to WT α_2_δ‐2, the mean peak amplitude was only approaching statistically significant difference from control neurons (Figure 8b–e). This suggests that homologous expression of α_2_δ‐2_R596P less efficiently enhances presynaptic Ca^2+^ transients than expression of WT α_2_δ‐2.

### 3.8 Trans‐synaptic signaling of α_2_δ‐2_R596P

**FIGURE 9:**
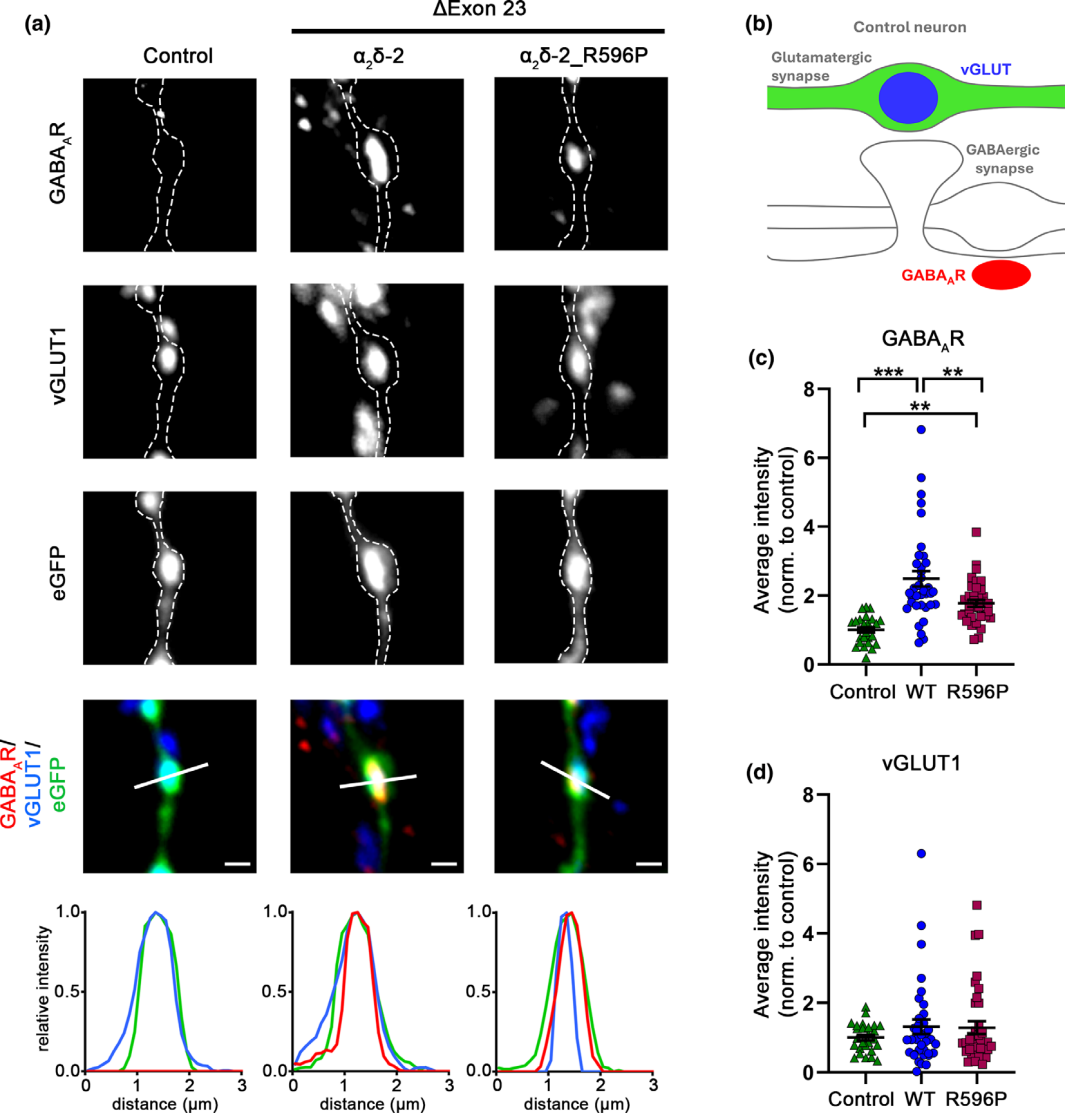
Reduced trans‐synaptic coupling to postsynaptic GABA_A_R of the α_2_δ‐2_R596P mutant. immunofluorescent labeling of presynaptic vGLUT1 and postsynaptic GABA_A_R was used to identify the formation of mismatched synapses in hippocampal neurons transfected with soluble eGFP as control or together with either WT or mutated α_2_δ‐2_ΔE23. (a) Both, homologous over‐expression of WT and mutated α_2_δ‐2 lead to the formation of mismatched synapses. However, compared to WT α_2_δ‐2, neurons transfected with α_2_δ‐2_R596P mutant showed a reduced postsynaptic GABA_A_Rs recruitment, as detected by postsynaptic GABA_A_R clusters opposite vGLUT1‐positive glutamatergic terminals (a, α_2_δ‐2, α_2_δ‐2_R596P). Colocalization of the fluorescence signals of representative images was analyzed using line scans. (b) Sketch summarizing the observed labeling pattern of a control bouton expressing eGFP alone. In control boutons, potential glutamatergic synapses are positive for presynaptic vGLUT1 (blue) but negative for postsynaptic GABA_A_Rs (red); in contrast to glutamatergic synapses, GABAergic synapses are typically located along the dendritic shaft. Quantifications of immunofluorescence intensities of GABA_A_R (c) and vGLUT1 (d) labeling show values for individual cells (dots) and means ± SEM (lines). Values were normalized to the fluorescent intensities of the control condition within each culture preparation. Cells were obtained from three independent culture preparations. **Statistics**: ANOVA with Tukey's multiple‐comparison test was performed on 26–37 cells per condition. GABA_A_R: *F*_(2,96)_ = 20.06; *p* < 0.0001. *P*‐values of the post hoc test for the respective pairwise comparisons: *P* < 0.0001 for control versus WT, *p* = 0.003 for control versus R596P, and *p* = 0.003 for WT versus R596P. vGLUT1: *F*_(2,96)_ = 0.81; *p* = 0.45. *P*‐values of the post hoc test for the respective pairwise comparisons: *P* = 0.47 for control versus WT, *p* = 0.53 for control versus R596P, and *p* = 0.99 for WT versus R596P. Significances of post hoc tests between conditions are indicated in the graphs by asterisks (***p < 0.001, **p < 0.01). Scale bars, 1 μm.

Presynaptic over‐expression of a splice variant of α_2_δ‐2 lacking exon 23 (E23) in hippocampal neurons has previously been shown to induce aberrant axonal wiring resulting in a mismatched localization of postsynaptic GABA_A_ receptors (GABA_A_R) opposite glutamatergic nerve terminals (Geisler et al., 2019). E23 is located downstream of R596 and codes for eight amino acids following residue 664. Structural homology modeling suggested that exclusion of this exon leads to the formation of an α‐helix (Geisler et al., 2019). To test whether introduction of the p.R596P mutation alters the trans‐synaptic function of α_2_δ‐2, we quantified the expression levels of postsynaptic GABA_A_R opposite mismatched vGLUT1‐containing presynaptic boutons upon homologous over‐expression of WT or mutated α_2_δ‐2_ΔE23 compared to neurons expressing eGFP alone as control. As shown previously (Ablinger et al., 2022; Geisler et al., 2019), WT α_2_δ‐2_ΔE23 induces clustering of postsynaptic GABA_A_R opposite vGLUT1‐positive terminals (Figure 9a,c, α_2_δ‐2 and WT, respectively). Neurons transfected with α_2_δ‐2_ΔE23_R596P were able to recruit GABA_A_Rs opposite glutamatergic nerve terminals (Figure 9a,c), however, GABA_A_Rs clustering was significantly reduced compared to WT α_2_δ‐2. This was particularly evident when quantifying the immunofluorescent signal of average fluorescence intensity of postsynaptic GABA_A_Rs. Because of the strong reduction in presynaptic expression of the R596P mutant (see above), a reduced trans‐synaptic action was to be expected. However, it is important to note that presynaptic expression is strongly reduced to 31% (confer Figure 6), while the trans‐synaptic recruitment of postsynaptic GABA_A_Rs is reduced to only 73% (confer Figure 9). Together this suggests that even small amounts and/or less stably expressed α_2_δ‐2 proteins can mediate a trans‐synaptic function.

**FIGURE 10:**
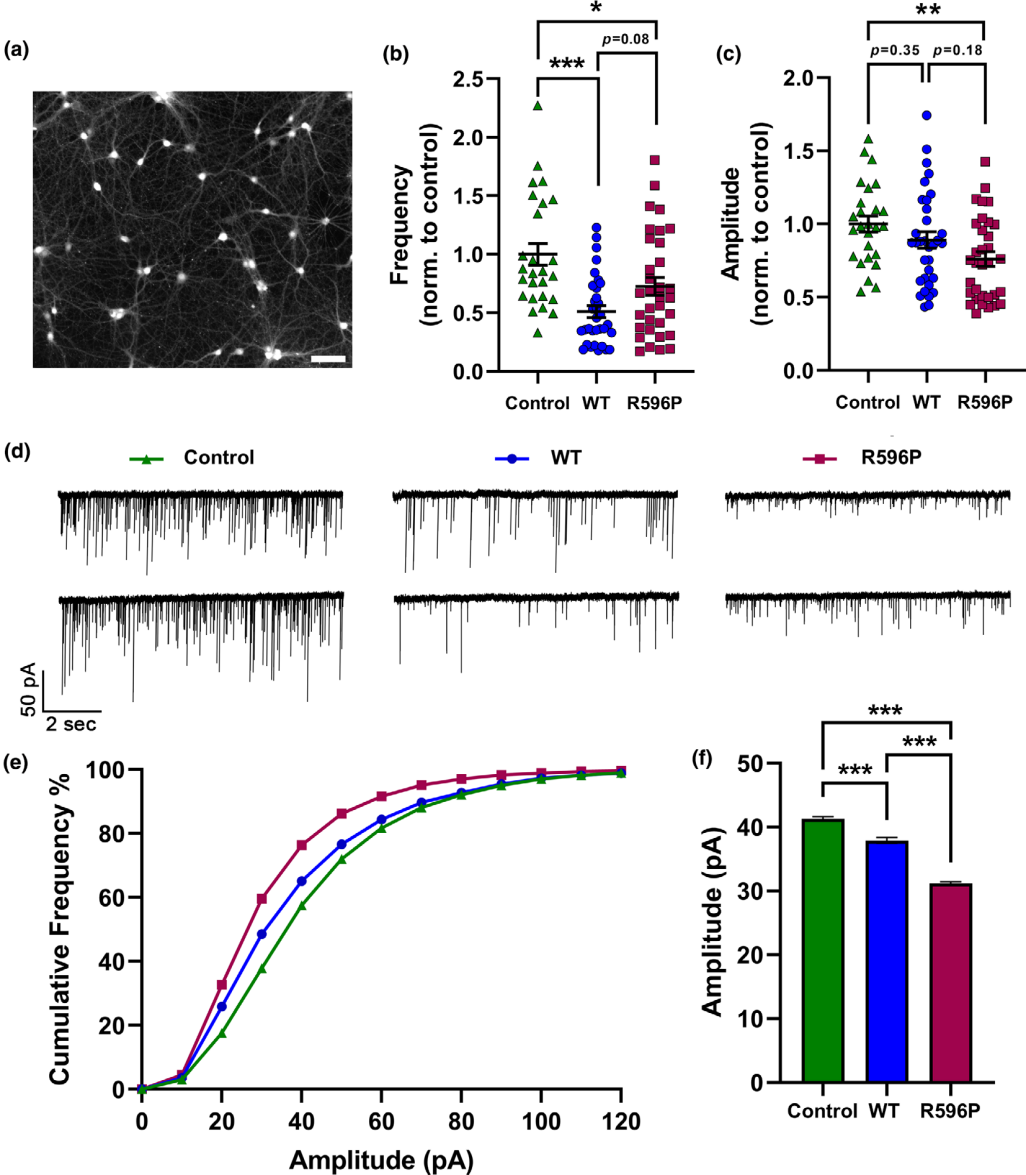
Homologous expression of α_2_δ‐2_R596P resulted in a greater reduction in spontaneous glutamate‐mediated synaptic transmission. Recordings of mEPSCs in 14–15 DIV cultured hippocampal neurons expressing soluble eGFP alone (control, green triangles), and either WT (WT, blue circles) or mutated (R596P, pink rectangles) α_2_δ‐2_ΔE23. (a) Representative image of hippocampal neurons at DIV 15 infected with α_2_δ‐2_R596P showing a ~ 100% infection efficiency. Scale bar, 200 μm. Quantification of mEPSC frequencies (b) and average amplitudes (c). Amplitudes and frequencies of mEPSCs were normalized to the mean value of control condition for each individual experiment. (d) Two representative traces of mEPSCs from independent recordings for all three conditions. (e) Cumulative frequency distribution histograms of all mEPSC amplitudes recorded from neurons shown in the previous panels. Number of analyzed events per condition: Control, *n* = 6012; WT, *n* = 3374; R596P, *n* = 5384. (f) Comparison of mean mEPSC amplitudes of all recorded events. Expression of the R596P mutant was associated with larger reduction in mEPSC amplitude compared to WT α_2_δ‐2. Statistics: (b, c) One‐way ANOVA with Tukey's multiple‐comparison test was performed on 26–33 recordings per condition from three independent culture preparations. (b) Frequency: *F*_(2,89)_ = 10.5; *p* < 0.0001. *P*‐values of the post hoc test for the respective pairwise comparisons: *P* < 0.001 for control versus WT, *p* = 0.03 for control versus R596P, and *p* = 0.08 for WT versus R596P. (c) Amplitude: *F*_(2,89)_ = 4.7; *p* = 0.01. P‐values of the post hoc test for the respective pairwise comparisons: *P* = 0.35 for control versus WT, *p* = 0.008 for control versus R596P, and *p* = 0.18 for WT versus R596P (f) One‐way ANOVA with Tukey's multiple‐comparison test was performed on 3374–6012 mEPSC events, *F*_(2,14767)_ = 273.6, *p* < 0.0001. *P*‐values of the post hoc test for the respective pairwise comparisons: *P* < 0.0001 for control versus WT, control versus R596P, and WT versus R596P. Significances of post hoc tests between conditions are indicated in the graphs by asterisks (***p < 0.001, **p < 0.01, *p = 0.031).

The aberrant wiring and postsynaptic GABA_A_R recruitment by presynaptic α_2_δ‐2_ΔE23 was associated with reduced glutamatergic synaptic transmission (Geisler et al., 2019). Hence, to further assess the trans‐synaptic consequences of α_2_δ‐2_R596P compared to WT α_2_δ‐2, we recorded and analyzed spontaneous miniature excitatory postsynaptic currents (mEPSCs). To ensure a uniform expression of the transfected α_2_δ‐2_ΔE23 cDNAs in all neurons (Figure 10a), we here used lentivirus‐mediated transfection. This is in contrast to the above‐mentioned experiments, in which liposomal‐mediated transfection was employed to obtain a low transfection efficiency of isolated presynaptic neurons. As expected for aberrantly wired synapses, both WT and mutated α_2_δ‐2 decreased the frequency of mEPSCs compared to neurons infected with eGFP‐expressing lentiviral particles only (control) (Figure 10b,d). However, consistent with the observed differences in postsynaptic GABA_A_R recruitment (confer Figure 9c), WT α_2_δ‐2 resulted in a larger decrease in mEPSCs frequency (on average reduction by ~50%; mean ± SEM: 0.51 ± 0.05) compared to the mutated α_2_δ‐2 (on average reduction by ~30%; mean ± SEM: 0.73 ± 0.08). An efficient and synchronized synaptic transmission requires precisely matched and aligned pre‐ and postsynaptic proteins. Hence, our data suggest that the strong reduction in mEPSC frequency is the result of a reduced number of functional synapses caused by the aberrant wiring of glutamatergic boutons with GABAergic postsynaptic sites. Interestingly, however, we also observed differences between the experimental conditions in the amplitude of the mEPSC, which is an indirect measure of postsynaptic AMPA receptor expression. The mean amplitude of mEPSCs recorded in neurons expressing mutated α_2_δ‐2 was significantly reduced by ~24%. In contrast, we did not observe a reduction in mEPSC amplitudes in neurons infected with WT α_2_δ‐2. Cumulative frequency distribution of mEPSC amplitudes (Figure 10e) and analysis of all individual mEPSC events (Figure 10f) show an even more prominent reduction of the mEPSC amplitude of neurons with R596P in comparison with control and WT α_2_δ‐2. Together, this shows that the decrease in mEPSC amplitude was not solely caused by altered wiring, but in the case of the mutated α_2_δ‐2 protein, it rather reflects an independent underlying mechanism.

## 4 DISCUSSION

Here, we provide a functional characterization of a potential disease‐causing CACNA2D2 mutation, which focuses not only on the Ca^2+^ channel‐dependent functions of α_2_δ‐2 but also on synaptic and trans‐synaptic functions of α_2_δ proteins. The p.R596P mutation decreases membrane expression and synaptic targeting of α_2_δ‐2. This defect in membrane expression differentially affects L‐type and non‐L‐type Ca^2+^ channels: while the p.R596P mutation alters current densities and voltage dependence of activation of the postsynaptic L‐type channel Ca_V_1.3 upon heterologous co‐expression, it has no effect on current properties of the presynaptic P/Q‐type channel Ca_V_2.1. However, in comparison with WT α_2_δ‐2 expression, α_2_δ‐2_R596P in hippocampal neurons did not significantly increase the presynaptic abundance of endogenously expressed Ca_V_2.1 channels and limited its capacity to increase presynaptic Ca^2+^ transients, even though there was no difference between the means of WT and R596P α_2_δ‐2. Despite the low membrane expression, α_2_δ‐2_R596P could still mediate trans‐synaptic signaling to postsynaptic GABA_A_Rs. Finally, homologous expression of mutated α_2_δ‐2 results in a defective presynaptic differentiation indicated by decreased presynaptic synapsin clustering, and altered postsynaptic differentiation indicated by reduced mEPSCs amplitudes.

### 4.1 R596 is critical for membrane expression of α_2_δ‐2

α_2_δ proteins, in their capacity as Ca^2+^ channel subunits, play an important role in membrane trafficking of the channel complex. However, α_2_δ proteins can also reach the cell surface independently of the VGCC complex (Canti et al., 2005; Cassidy et al., 2014; Schredelseker et al., 2005). Structural homology modeling of α_2_δ‐2 predicts one critical interaction of the highly conserved R596 with Y1094 within the δ peptide. Hence, R596 is predicted to maintain the interaction between α_2_ and δ peptides and thereby stabilize the whole structure of α_2_δ‐2. Indeed, α_2_δ‐2_R596P shows a reduced membrane expression both upon heterologous and homologous expression. Correct folding of membrane proteins in the endoplasmic reticulum (ER) following translation is crucial for the forward trafficking to the cell surface and aberrantly folded proteins are retained in the ER and may be subject to degradation (Vembar & Brodsky, 2008). However, this seems not to be the case for α_2_δ‐2_R596P as total protein expression was not compromised by the mutation. Hence, the reduced membrane expression suggests that the p.R596P mutation either affects forward trafficking from the ER and/or increases the internalization rate caused by destabilization of the protein on the cell surface.

### 4.2 The p.R596P mutation uncovers isoform‐ and cell‐type‐specific differences in α_2_δ‐2‐mediated functions

All α_2_δ isoforms augment the Ca^2+^ currents of all Ca_V_1 and Ca_V_2 channels with little effect on the single‐channel conductance (Barclay et al., 2001; Tuluc et al., 2007), implying that α_2_δ proteins mediate the current enhancement by mainly increasing the functional membrane expression of the channel complexes. While the exact underlying mechanisms remain elusive, a crucial role of the MIDAS motif within the VWA domain in membrane trafficking of the α_1_ subunit seems likely (Canti et al., 2005). Our present data show that p.R596P differently affects the ability of α_2_δ‐2 to enhance Ca^2+^ currents of L‐type (Ca_V_1) and non‐L‐type (Ca_V_2) channels upon heterologous co‐expression. While the p.R596P mutation significantly reduced the current density and shifted the voltage dependence of activation to more depolarized potentials of Ca_V_1.3 channels, it did not affect the biophysical properties of Ca_V_2.1 channels. This differential effect could be attributed to two mechanisms: differences in the affinity between α_2_δ‐2 and α_1_ isoforms (Voigt et al., 2016) or membrane expression ceiling effects of Ca_V_2 channels (Scott & Kammermeier, 2017). The fact that Ca_V_2.1 α_1_, α_2_δ‐2, and β_4_ are the dominating isoforms expressed in the cerebellum and that their expression levels co‐increase during neuronal differentiation (Schlick et al., 2010) indicates endogenous complexing of these subunits. We can speculate that in contrast to Ca_V_1.3, the affinity between Ca_V_2.1 and α_2_δ‐2 could be strong enough to tolerate the p.R596P mutation as well as its reduced membrane expression. As Ca_V_2 channels possess an upper limit of membrane expression (Cao et al., 2004; Scott & Kammermeier, 2017), it seems more likely that slight changes in current densities may be masked by this property. To test this, we reduced the amount of transfected α_2_δ‐2 WT and mutated cDNA by fivefold. However, also under this experimental paradigm, co‐expression of α_2_δ‐2_R596P did not reduce the current density of Ca_V_2.1 channels. This finding suggests that the mutation does not impede membrane trafficking of Ca_V_2.1 channels in a heterologous expression system, and hence, it differentially affects L‐type and non‐L‐type Ca^2+^ channels.

Because surface expression of α_2_δ‐2_R596P was also strongly reduced in hippocampal neurons, we next tested the consequence of the mutation on endogenous Ca_V_2.1 channels in their native neuronal environment. An increase in presynaptic Ca_V_2.1 clustering and presynaptic Ca^2+^ transients is a hallmark of homologous expression of α_2_δ isoforms in wild‐type hippocampal neurons (Ablinger et al., 2022; Schopf et al., 2021). In contrast to heterologous co‐expression, α_2_δ‐2_R596P did not increase presynaptic clustering of endogenous Ca_V_2.1 channels and presynaptic Ca^2+^ transients to the same extent as WT α_2_δ‐2, whereby the means of WT and mutated α_2_δ‐2 were not significantly different. Altered modulation of endogenous Ca_V_2.1 by mutated α_2_δ‐2 in synapses of differentiated hippocampal neurons uncovers cell‐type‐specific modulation by α_2_δ proteins which cannot be reconstituted in a heterologous expression system. Together, this suggests that modulation of Ca^2+^ channels by α_2_δ‐2 proteins is not limited to α_1_/α_2_δ‐2 protein–protein interactions but also involves the local macromolecular environment of a specialized cellular compartment such as the presynaptic terminal. Moreover, this finding highlights the importance of studying the mechanistic consequences of disease‐associated mutations within their specific and native cellular environment.

### 4.3 The p.R596P mutation affects pre‐ and postsynaptic differentiation by distinct mechanisms

α_2_δ‐2_R596P showed a reduced, albeit still functional, trans‐synaptic recruitment of postsynaptic GABA_A_R, a recently identified trans‐synaptic function of α_2_δ‐1 and α_2_δ‐2 splice variants in both GABAergic and glutamatergic synapses (Ablinger et al., 2022; Geisler et al., 2019). It is still elusive whether this trans‐synaptic function of α_2_δ is mediated by a direct trans‐synaptic interaction or indirectly by the interaction with other synaptic organizers. On the one hand, the reduced trans‐synaptic function of α_2_δ‐2_R596P together with its reduced surface expression point toward a direct trans‐synaptic interaction. On the other hand, the fact that the effect on trans‐synaptic GABA_A_R recruitment is much smaller than the reduction in presynaptic membrane expression of α_2_δ‐2_R596P indicates that even small amounts and/or less stably expressed α_2_δ‐2 proteins are sufficient for trans‐synaptic signaling. Even though the trans‐synaptic recruitment of GABA_A_Rs and its consequence on synaptic transmission were observed in a non‐physiologic experimental paradigm, namely the over‐expression in glutamatergic neurons, it may provide important insights into potential physiological roles of α_2_δ‐2. Purkinje cells have a single long axon that forms an inhibitory projection to the cerebellar nuclei (Paul & Limaiem, 2023) and they strongly express α_2_δ‐2 mRNA (Barclay et al., 2001). Hence, modulating the trans‐synaptic signaling with postsynaptic GABA_A_R, for example, by the p.R596P mutation of α_2_δ‐2, may lead to a decreased functionality of this inhibitory GABAergic projection. Subsequently, this can lead to an excitatory–inhibitory imbalance which may ultimately underlie the epileptic phenotype observed in the patients.

Recent findings suggest a pivotal and highly redundant role of presynaptic α_2_δ proteins in the formation and differentiation of glutamatergic synapses in CNS neurons (Ablinger et al., 2022; Schopf et al., 2021). Our present data show that expression of α_2_δ‐2_R596P in WT neurons endogenously expressing three α_2_δ isoforms (α_2_δ‐1‐3) affects pre‐ and postsynaptic differentiation as indicated by reduced presynaptic synapsin clustering and smaller mEPSC amplitudes. This phenotype, although milder, resembles the defect in synaptic differentiation observed in α_2_δ triple‐knockout/knockdown neurons (Schopf et al., 2021). α_2_δ proteins homologously expressed in hippocampal neurons must compete with endogenously expressed α_2_δ isoforms. Hence, the altered consequences of expressing mutated α_2_δ‐2 in comparison with WT α_2_δ‐2, such as defective pre‐ and subsequently postsynaptic differentiation, indicate a dominant‐negative effect. Even more so as total neuronal surface expression is strongly affected by the p.R596P mutation. A similar situation may occur in the patients harboring the recessive p.R596P mutation: since α_2_δ‐2 is the dominating cerebellar isoform and both patients were found to be homozygous for the mutation, synapse differentiation may be affected by the dominant negative behavior of the mutated α_2_δ‐2 protein in competition with the remaining endogenous α_2_δ isoforms 1 and 3.

The negative effect of homologous α_2_δ‐2_R596P expression on the amplitudes of mEPSCs is an interesting observation by itself. The reduced synapsin content upon expression of mutated α_2_δ‐2 may indicate a defect in presynaptic vesicle recruitment and hence may contribute to a smaller amount of neurotransmitter release in response to spontaneous Ca^2+^ influx, which leads to a reduced response of postsynaptic AMPA receptor. Alternatively and given the pivotal role of presynaptic α_2_δ for both pre‐ and postsynaptic differentiation and the identified trans‐synaptic functions of α_2_δ isoforms (Ablinger et al., 2022; Fell et al., 2016; Geisler et al., 2019), p.R596P may exert its deleterious effect via defective trans‐synaptic signaling thereby changing the abundance of postsynaptic AMPA receptors.

Taken together, our data demonstrate three consequences of the p.R596P mutation on synaptic functions, which may only be partially related: first, reduced trans‐synaptic recruitment of GABA_A_R; second, reduced presynaptic synapsin clustering in glutamatergic nerve terminals; and third, reduced amplitudes of mEPSCs.

### 4.4 Potential disease mechanisms

The functional characterization of the bi‐allelic mutation p.R596P in α_2_δ‐2 provides three possible mechanistic explanations for how a defective α_2_δ‐2 protein could be linked to DEE. Firstly, mutations in α_2_δ‐2 can affect its role as a VGCC subunit and disrupt channel‐dependent functions in an isoform‐ (differential effects on Ca_V_1.3 L‐type and Ca_V_2.1 P/Q‐type channels) and tissue‐specific (presynaptic abundance of Ca_V_2.1) manner. Either way, normal Ca^2+^ handling in pre‐ and postsynaptic compartments will be affected. Secondly, the reduced trans‐synaptic coupling to postsynaptic GABA_A_Rs accompanying the low membrane expression of α_2_δ‐2_R596P may reduce the functionality of GABAergic inhibitory synapses, which is of relevance for the predominant expression of α_2_δ‐2 in the cerebellum. Thirdly, the reduction in presynaptic clustering of synapsin and the amplitude of mEPSCs are indications of aberrant synaptic differentiation which decreases the probability of neurotransmitter release and weakens synaptic connectivity. All these proposed pathophysiological mechanisms may ultimately lead to an aberrant excitatory–inhibitory balance that underlies DEEs and, more broadly, neurodevelopmental disorders. Moreover, our study emphasizes the importance of studying the consequences of disease‐associated α_2_δ protein mutations on both the classical functions as VGCC subunits and synaptic functions.

## AUTHOR CONTRIBUTIONS

**Sabrin Haddad:** Funding acquisition; investigation; conceptualization; writing – original draft; methodology; writing – review and editing. **Cornelia Ablinger:** Software; conceptualization; methodology; writing – review and editing; investigation. **Ruslan Stanika:** Conceptualization; investigation; methodology; writing – review and editing; supervision. **Manuel Hessenberger:** Conceptualization; investigation; methodology; visualization; writing – review and editing; supervision. **Marta Campiglio:** Investigation; methodology; writing – review and editing; supervision. **Nadine J. Ortner:** Supervision; methodology; conceptualization; writing – review and editing. **Petronel Tuluc:** Conceptualization; supervision; writing – review and editing; methodology. **Gerald J. Obermair:** Conceptualization; funding acquisition; writing – review and editing; writing – original draft; methodology; project administration; supervision; resources.

## CONFLICT OF INTEREST STATEMENT

The authors declare no competing financial interests.

### PEER REVIEW

The peer review history for this article is available at https://www.webofscience.com/api/gateway/wos/peer‐review/10.1111/jnc.16197.

## Data Availability

Not applicable or already included in the manuscript.
